# Concurrent photocatalytic degradation of organic pollutants using smart magnetically cellulose-based metal organic framework nanocomposite

**DOI:** 10.1038/s41598-025-03256-5

**Published:** 2025-06-20

**Authors:** Nora A El-Mahdy, Sayed RH El-Gharkawy, Magda A Akl

**Affiliations:** Chemistry Department, Faculty of Science, Mansoura University, Mansoura, 35516 Egypt

**Keywords:** Magnetic cellulose-based nanocomposite, MOF, Photocatalytic degradation, computational studies, Cationic and anionic dyes, error functions, Analytical chemistry, Chemical biology, Chemical engineering, Environmental chemistry

## Abstract

Industrial activities, especially textiles and cosmetics, release harmful wastewater, threatening the environment and human health. Photocatalysis has emerged as an effective, eco-friendly solution for these issues, particularly using metal-organic frameworks (MOFs) for water treatment. This study explores the performance, computational analysis, and mechanistic behavior of a novel magnetically responsive cellulose-based metal-organic framework (MOF) nanocomposite, DAC@PdA@FM, for the simultaneous photocatalytic degradation of Toluidine Blue O (TBO), Crystal Violet (CV), and Sunset Yellow FCF (E110) dyes. The material was synthesized using a controlled oxidation method and characterized using FTIR, XRD, EDX, SEM, TGA techniques and PPPS saturation magnetization properties. The uptake capacity of DAC@PdA@FM toward organic dyes as TBO,  CV, and E110 from water, achieving reductions of 988.75, 1242.5, and 497 mg/g, respectively, within short time frames.The kinetic and isotherm studies were best fitted by PSO and the Langmuir models due to the higher correlation coefficient (R^2^ ≥ 0.999) and the lower error functions. The nanocomposite exhibited enhanced reusability and separation efficiency due to its superparamagnetic nature. Density functional theory (DFT) calculations confirmed the electronic structure and charge transfer mechanisms. Comparative analysis with previous studies confirmed superior degradation efficiency. The results also suggest that the MOF: DAC@PdA@FM nanocomposite possesses notable antimicrobial activity,  particularly against gram-ve bacteria. These findings suggest that the MOF: DAC@PdA@FM nanocomposite is a promising applicant for wastewater treatment applications. The catalytic degradation mechanism for dyes on the prepared MOF:DAC@PdA@FM nanocomposite involves various interactions, including electrostatic attraction, pore-filling, π–π stacking, and hydrogen bonding. Also, The results suggest that utilizing pre-prepared MOF:DAC@PdA@FM nanocomposite could serve as a potent and efficient antimicrobial agent.

**Supplementary Information:**

The online version contains supplementary material available at 10.1038/s41598-025-03256-5.

## Introduction

Freshwater scarcity is a significant global challenge^1,2^, affecting two to three billion people for at least one month annually, according to the UN’s World Water Development Report 2022^3^. Additionally, industrial synthetic chemicals, particularly from the textile sector, contribute to water pollution, harming aquatic ecosystems by discoloring water and diminishing light penetration. Also, if chemicals are improperly removed, they can leach into the soil, affecting soil fertility and potential groundwater. Chemical pollutants come from various sources, including industrial, agricultural, and urban runoff. Major groups include pesticides and herbicides, commonly found in agriculture and residential areas, such as Atrazine and glyphosate^4^. These pollutants contaminate water sources, harm aquatic life and human health. Industrial chemicals, like PCBs and dioxins, arise from industrial processes and persist in the environment, leading to severe long-term health effects, including cancer. Pharmaceuticals and personal care products (PPCPs), often released from wastewater and improper disposal, such as antibiotics, can disrupt ecosystems and contribute to antibiotic resistance. Nutrients, primarily from agricultural practices, like nitrate and phosphate, can lead to excessive growth of algae, causing oxygen-depleted “dead zones“^5^. Volatile organic compounds (VOCs), emitted from chemicals and fuel combustion, such as benzene, are flammable and pose additional environmental risks. Moreover, one of the primary sources of aromatic pollutants in industrial wastewater is Organic dyes^6^.

Dyes can release volatile organic compounds (VOCs) and other pollutants into the air, contributing to air pollution and potential health hazards to nearby communities known or suspected that certain chemicals and substances a breakdown can cause cancer or have other adverse health effects in humans and animals^7^. Among these dyes, Toluidine Blue O (TBO) is a chemical commonly used in tissues and cells to stain acidic polysaccharides, mucins, and cartilage. TBO can be harmful if inhaled, ingested, or in contact with the skin or eyes with possible irritation, blistered, and poisoned in high doses^8^. Whereas crystal violet dye (CV) is beneficial, it may be just as hazardous and harmful as TBO if not handled correctly^9,10^. Sunset Yellow FCF, often commercially known as E110, is a pigment used in food as well as cosmetics. Although most people believe tiny amounts to be harmless, others are concerned about potential negative effects. Some individuals may become sensitive or allergic to E110, causing symptoms such as nausea, pain and asthma. Furthermore, it can cause cancer in humans. Consequently, the release of synthetic chemicals from waste streams has become a major environmental concern because even small amounts of chemicals can be highly toxic and detectable^11^.

Traditional water treatment methods, like desalination^12^, reverse osmosis^13^, nano filtration and electro dialysis, vapor compression^14–16^, and distillation^17^, are often costly and energy-intensive. Thus, researchers are exploring alternative resources, such as atmospheric water vapor. Various energy-efficient methods have been proposed for extracting this resource, including the use of hygroscopic materials, cooling air below dew point, fog harvesting, and advanced technologies like green roofs and hydrogels.

Scientists being aware of the harmful nature of dyes, have worked hard to break this barrier. Wherefore, they have developed various physical, chemical and biochemical properties wastewater treatment methods of these processes including ion-exchange^18^, flocculation^19^, membrane separation^20^, biodegradation coagulation^21^, electrochemical process^22^, adsorption^23–25^, advanced oxidation processes^26^, and photo catalysis^27,28^.

Photocatalytic degradation is an advanced oxidation process in which light (usually UV light or visible light) acts as a photocatalyst, leading to the formation of reactive species such as hydroxyl radicals that break down pollutants carbon in air and water. The process of photocatalysis, which involves the use of light and oxygen to break down organic and inorganic wastes, can result in the production of non-toxic by-products like carbon dioxide and water. This environmentally friendly method works in simple conditions and is energy efficient. Photo catalysts can be reused many times, which contributes to the stability and effectiveness of the process^29,30^. Absorbents are materials like sponges that absorb substances, while adsorbents, such as activated carbon, trap substances on their surface. Ion exchange sorbents, like ion exchange resins, replace unwanted ions in solutions. Natural materials, including bentonite clay^31^, effectively absorb oils and heavy metals, whereas synthetic polymer sorbents^32^ target specific contaminants. Bio-based sorbents, such as chitosan^33^, provide eco-friendly alternatives, and activated alumina^34^ is used to remove contaminants like fluoride from water. Metal-organic frameworks (MOFs) are porous materials that aid in gas capture and water purification^35^. Among these, metal organic frameworks (MOFs) and coordination polymers offer promising potential for addressing freshwater demand. Both are porous crystalline materials that have garnered significant attention in various scientific and industrial fields, including adsorption processes^36^. Both materials have structural and functional advantages that make them more useful for their ability to accommodate gas storage^37^, selective catalytic reactions^38^, and sensor applications^39^. MOFs consist of groups or clusters of metal ions coordinated with organic ligands to form one-dimensional, two-dimensional, or three-dimensional structures. The combination of metallic junctions and organic binders provides highly porous materials with tunable pore size characteristics^40,41^.

Thousands of MOFs have been synthesized, each with unique properties tailored for specific applications. MOFs can be performed a specific surface of more than 7000 m^2/^g, offers a wide range of application areas. MOFs are capable of adsorbing a wide range of molecules, including gases (e.g. CO_2_, CH_4_), liquid-phase pollutants^42^, volatile organic compounds (VOCs) and can be externally modified for selectivity toward specific functional groups^43^. They also maintain structural integrity under diverse thermal and chemical conditions, enhancing their suitability for numerous applications. Consequently, some are used for heavy metal removal as ZIF-8^44^, ZIF-67^45^ and arsenic on MIL-53(Al)^46^. Also, others are utilized like MIL-101(Fe)^47^ in the advanced catalytic Oxidation, UiO-66^48^, UiO-67^49^ and HKUST-1 (Cu-BTC)^50^ in the adsorption of dyes, pesticides and (VOCs), respectively, Fe-Cu Mixed-Metal MOFs in the degradation of organic dyes^51^ and MOF composites for enhancing adsorption properties^52^. The inclusion of a MOF component in the composite not only enhanced its structural and photocatalytic properties but also contributed to its antimicrobial potential, as MOFs are known to exhibit intrinsic antibacterial activity due to their high surface area, metal ion content, and reactive functional groups^53^.

Cellulose-based materials have attracted growing interest in photocatalytic applications due to their biodegradability, renewability, and excellent surface modification capabilities. By integrating cellulose with MOFs and magnetic nanoparticles, it is possible to develop hybrid nanocomposites with enhanced adsorption, catalytic efficiency, and easy recyclability. Recent studies have explored hybrid materials and MOF composites for dye removal, highlighting the importance of magnetic recovery and multifunctional performance in water purification systems^52,53^.

Herein, we synthesized a magnetically recoverable cellulose-based MOF nanocomposite (DAC@PdA@FM) and evaluated its potential for the simultaneous degradation of cationic (Toluidine Blue O, Crystal Violet) and anionic (Sunset Yellow FCF, E110) dyes.

The novelty of this work lies in the fabrication of a cellulose-derived MOF composite with magnetic properties, enabling efficient separation and reuse. The study investigates the composite’s physicochemical characteristics, adsorption and photocatalytic degradation performance, reaction kinetics, thermodynamics, and computational insights. The results provide a deeper understanding of the fundamental degradation mechanisms and potential applications in real- wastewater treatment.

Based upon the above-mentioned information, the objectives of this study were (i) Fabrication of the MOF nanocomposite with magnetic properties(DAC@PdA@FM) (ii) to investigate the physico-chemical properties of the prepared DAC@PdA@FM using various instruments like FT-IR, TGA, SEM, XRD, and EDS and magnetization (iii) to investigate the effect of different parameters during the photocatalytic degradation of the studied cationic and anionic dyes like pH, dosage, initial dye concentration, time, and co-existing ions; (iv) Statistical analysis of the isotherm and kinetic models using the chi-square statistic (χ^2^), mean square error (MSE), the sum of squares error (SSE), and Hybrid error; v. Comparative evaluation of dye removal efficiency (RE), feasibility, and reusability of the MOF: DAC@PdA@FM nanocomposite with other photocatalysts; (vi) to investigate the biological activity of the novel MOF: DAC@PdA:FM nanocomposite; (vii) to investigate the molecular and electronic properties, spectral properties, and some quantum mechanical calculations of DAC@PdA@FM, DAC@PdA@FM-TBO, DAC@PdA@FM-CV, DAC@PdA@FM-E110 and (viii) Elucidation of the mechanisms involved in the processes of photodegradation of dyes using the MOF: DAC@PdA@FM nanocomposite. Moreover, the MOF: DAC@PdA@FM nanocomposite was also tested in real water samples.

## Experimental

### Materials

Cellulose powder (microgranular, C6413) was used as the primary polysaccharide for composite synthesis.The molecular weight of cellulose’s repeating unit is 162.14 g/mol. 1, 2-phenylenediamine(PdA) (99.5%), potassium periodate (KIO_4_(99.8%)), potassium hydroxide (KOH), iron (III) chloride hexahydrate (FeCl_3_.6H_2_O), ammonium hydroxide (25%), Iron (II) sulfate heptahydrate (FeSO_4_.7H_2_O), purified Terephthalic acid (TPA), HCl, NaOH(97%), Conc. H_2_SO_4_, Na_2_CO_3_, NaCl, ethanol, N, N-dimethylformamide (DMF), Toluidine blue O (TBO), Crystal violet (CV) as cationic dyes and (E110) as anionic dye. 4 g of KIO_4_ were dissolved in 100 mL dist.H_2_O for preparing 4% potassium periodate solution. All chemicals were used without any further purification and were obtained from Sigma Aldrich. The classifications, chemical structures and absorption wavelengths of the used organic dyes and Terephethalic acid (TPA) are shown in Table 1.

**Table 1:** The classifications, chemical structures and absorption wavelengths of some commonly used organic dyes and terephthalic acid (TPA).

Name of dye	color	Chemical structure	Chemical Formula	Nature	Absorption λmax (nm)
Toluidine blue O			C_15_H_16_N_3_S^+^	Cationic	630
Crystal violet			C_25_H_30_ClN_3_	Cationic	590
E110			C_16_H_10_N_2_Na_2_O_7_S_2_	Anionic	485
**Terephthalic acid (TPA)**					
**IUPAC NAME**		Benzene-1,4-dicarboxylic acid			
**Chemical structure**					

### Characterization

A 150 W Xenon lamp (6256 Newport) emitting light in the 320–400 nm UV range with an intensity of 100 mW cm^−2^ was used for the photocatalytic degradation of TBO, CV, and E110 dyes. Analysis of the prepared nanocompositeincluded Fourier Transform Infrared (FTIR) spectroscopy at a wavenumber range of 4000–400 cm^−1^, Scanning electron microscopy (SEM) for surface morphology, and Energy Dispersive X-ray Spectroscopy (EDS) for elemental composition of the DAC@PdA@FM nanocomposite. X-ray diffraction (XRD) patterns were obtained for the composites using a PAN analytical X’Pert PRO diffractometer, and thermal stability was assessed through thermogravimetric analysis (TGA) from 30 to 800 °C. The CHN composition of native cellulose and DAC@PdA was determined with a Costech ECS-4010 elemental analyzer, while UV-Vis spectra for Nano ferromagnetite (Fe_3_O_4_) were collected using a PerkinElmer 550 spectrophotometer in ethanol.The saturation magnetization (MS) values of MOF: DAC@PdA@FM nanocomposite was determined by the Cryogenic Limited PPMS (Physical Property Measurement System) at room temperature. A Neodymium N52 magnet (Dimensions: 50 × 20 × 7 mm, pulling force: 15 kg (was used to externally separate the MOF: DAC@PdA@FM loaded dyes after the sorption photocatalytic degradation of dyes. The photocatalytic degradation studies were achieved via a Thermo Scientific Evo 201 Double Beam UV-VIS Spectrophotometer to determine the yields.

The pH_PZC_ of the MOF: DAC@PdA@FM nanocomposite was determined by mixing 0.1 g of the composite with 25 mL of 0.01 M NaCl solutions at varying pH levels (2–12) for 48 h. The pH of the NaCl solutions was adjusted using 0.1 M HCl and 0.1 M NaOH. After shaking, the final pH was recorded, and ΔpH was calculated (ΔpH = pHi – pHf). The pHPZC was identified as the point where ΔpH equals 0 on the ΔpH vs. initial pH (pHi) plot^24,54^.

### Preparations

#### Synthesis of dialdehyde cellulose (DAC) and the aldehyde content (%) assessment

**Fig. 1:**
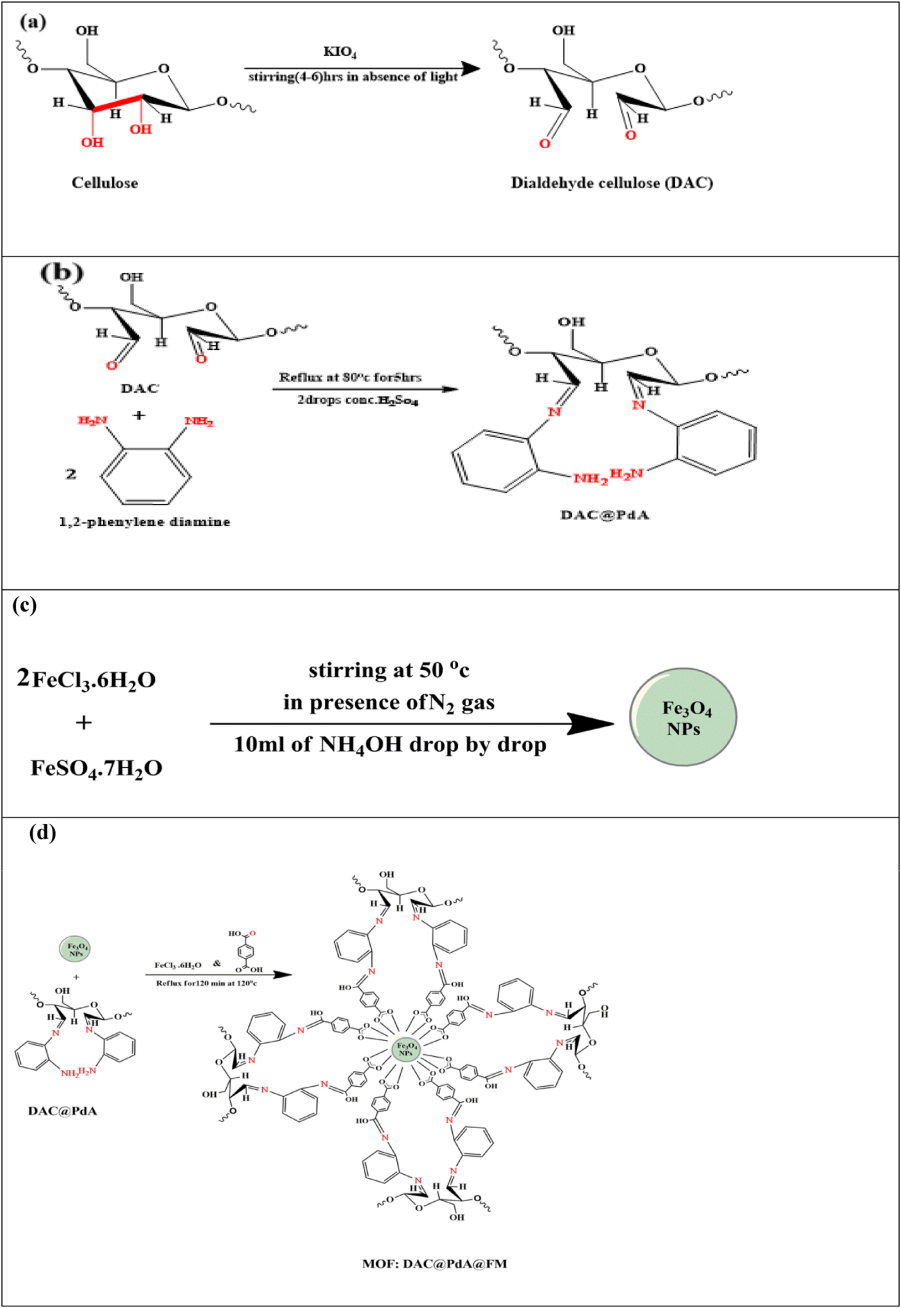
Synthesis of (**a**) 2, 3 dialdehyde cellulose (DAC), (**b**)PdA modified dialdehyde cellulose (DAC@PdA), (**c**) magnetite nanoparticles (Fe_3_O_4_) and (**d**) magnetic metal–organic framework nanocomposite (MOF: DAC@PdA@FM).

The oxidation process of one gram cellulose occurred using 4% KIO_4_ (100 mL), in the whole absence of light to prevent extensive oxidative degradation with the formation of free radicals and modification of functional groups, as shown in Fig. 1. The formed mixture was stirred for 6 h at 50^o^c. The prepared oxidized cellulose (DAC) was filtered, washed several times with water and ethanol, then finally dried in an oven at 50 °C. The aldehyde content (AC %) of the prepared DAC was determined by mixing 0.1 g of DAC with 25 mL of 250 mM hydroxylamine HCl at pH 4.0, stirring for 2.5 h in darkness. The DAC was then filtered, dried at 70 °C, and the filtrate back titrated with 0.1 M NaOH to pH 4.0, indicating the endpoint when the color changed from red to yellow^55^. The prepared DAC is illustrated in Fig. 1a. The DAC aldehyde content percentage (AC (%)) was determined using Eq. (1), where V sample and V control represent the volumes of NaOH for oxidized cellulose and native cellulose powder, respectively. Additionally, the sample weight is represented by m, and M.wt stands for the molecular weight of cellulose.

1 $$\:\text{A}\text{C}\text{\%}=\frac{\text{N}\text{a}\text{O}\text{H}\:\text{c}\text{o}\text{n}\text{c}\text{e}\text{n}\text{t}\text{r}\text{a}\text{t}\text{i}\text{o}\text{n}\:\left(\text{V}\text{s}\text{a}\text{m}\text{p}\text{l}\text{e}-\text{V}\text{c}\text{o}\text{n}\text{t}\text{r}\text{o}\text{l}\right)}{\raisebox{1ex}{$\text{m}$}\!\left/\:\!\raisebox{-1ex}{$\text{M}.\text{W}\text{t}$}\right.}$$

#### Preparation of 1, 2-phenelyne Diamine (PdA) modified dialdehyde cellulose (DAC@PdA)

The DAC@PdA produced from the refluxing of the oxidized cellulose (0.9621 g) dissolved in 20 mL ethanol with 1, 2-phenelyne diamine **(**PdA) (0.6512 g) dissolved in 10 mL ethanol in presence of 2 drops of conc.H_2_SO_4_ for 6 h. At 75 ^o^ C according to reaction synthesis, Fig. 1b. During the reaction, the color of the prepared mixture was changed to a formed reddish-brown precipitate. This formed mixture was filtered off, washed several times with water and ethanol, and after ward dried in oven at 50 ^o^ C.

#### Preparation of magnetite nanoparticles (Fe_3_O_4_)

Firstly, FeCl_3_·6H_2_O and FeSO_4_.7H_2_O with molar proportion of 2:1 was dissolved in 100 mL deionized water in a round flask under N_2_ gas for 30 min. And then 10 mL of ammonium hydroxide (1 M) was added drop by drop. The mixture was stirred for 10 min at 1000 rpm under constant magnetic stirring, directly a black precipitate appeared, which is separated by applying an external magnetic field. Finally, the product was washed several times with deionized and water and Ethanol then dried at 120 °C for 2 h. The preparation of Nano Fe_3_O_4_ is illustrated in Fig. 1c.

#### **Synthesis of magnetic metal–organic framework nanocomposite** (**MOF**: **DAC@PdA@FM**)

The MOF: DAC@PdA @FM nanoparticles were effectively synthesized according to^56^with some modifications. The preparation of MOF: DAC@PdA@FM nanocomposite mainly involved three steps: firstly, 0.2 g of DAC@PdA with 0.2 g of nano magnetite (Fe_3_O_4_)were dissolved in dimethylformamide (DMF) with ethanol under the effect of vigorous stirring followed by sonication process for 30 min at 60^◦^C until complete dissolution to avoid aggregation. Then, 3 mmol of FeCl_3_·6H_2_O dissolved in 10 mL absolute alcohol and 2 mmol of PTA dissolved in 10 mL DMF. This mixture was added dropwise to 30 mL solution of 0.2 g of DAC@PdA with 0.2 g of Nano magnetite Fe_3_O_4_ at room temperature. Finally, the resultant mixture was condensed with a stirring rate of 450 rpm for 360 min at 120 ^o^c. Eventually, the resulting MOF: DAC@PdA@FM composite was centrifuged and washed by deionized water and absolute alcohol for several times, then dried at 120 ^◦^C for 3 h in an oven. The synthesis steps of MOF: DAC@PdA@FM nanocomposite is illustrated in Fig. 1d.

### Photocatalytic degradation procedures

The photocatalytic tests were carried out in a batch catalytic system. The data are presented on the simple mean of three replicates, whereas those of applications were repeated five times from which the statistical analysis could be achieved. A 150 W Xenon lamp (6256 Newport) with a wavelength range of 400–700 nm and an intensity of 100 mW cm^−2^ was employed for the photodegradation of dyes. The lamp was placed vertically in the reaction system. To perform the photocatalytic activity experiment, the synthesized photocatalyst was added into an aqueous dyes’ solution (TBO, CV and E110) ranged from (50–300 ppm). For dye mixture degradation experiment, the 3 dyes were mixed: 200 ppm TBO, 250ppm CV and 100 ppm E110. A series of degradation experiments were conducted using 125 mL transparent bottles. First, the suspension was placed in dark for an hour to ensure it reaches the adsorption equilibrium before turning on the lamp. The initial adsorption phase resulted in less than 15% removal.Then, the Xenon lamp was switched and the experiments were carried out in certain moments. The distance between the solution and the light source fixed at 10.0 cm during photodegradation. Every 15 min the dispersed photocatalysts were easily collected with an external magnet and the concentration of the residual dye in the solution was monitored using UV–visible spectroscopy within the spectral range of 200–800 nm. To prepare the Fe_3_O_4_: DAC@PdA@FM composite solution, dissolve a small amount in distilled water and stir it well. Allow the particles to settle for a minute. Introduce a neodymium magnet near the waterline of the beaker, attracting the magnetic MOF particles to one side. With the magnet in place, pour off the clear water or carefully use a pipette to remove it, keeping the clustered particles intact. If any particles remain dispersed, repeat the magnet movement. The use of a neodymium magnet in this step was essential to verify the magnetic responsiveness of the Fe₃O₄: DAC@PdA@FM nanocomposite. Its strong field enabled rapid and complete recovery of the photocatalyst from solution, demonstrating its potential for practical magnetic separation without the need for filtration or centrifugation. For the Binary system, The tested Solutions containing a mixture of CV, TBO and E110 were prepared at optimal conditions of concentration, volume and dosage for each dye. The appropriate pH of each mixture was adjusted by diluted HCl and NaOH before photocatalytic reduction process^57^.

The degradation performance of dye in each test and the reaction rate constant were obtained based on Eqs. (2, 3)

2 $$\:\%{D}_{e}=\left(1-\frac{C}{{C}_{o}}\right)\times\:100$$

3 $$\:-\text{ln}\left(C/{C}_{o}\right)=Kt$$

The percentage of degradation efficiency, D_e_, for the photocatalytic reaction was calculated by Eq. 2, where C_0_ and C are the dyes concentrations at reaction times 0 and t, respectively. The reaction rate constant has been calculated for the photocatalyst nanoparticles by Eq. 3, where k illustrates the first-order rate constant; C and C_0_ show the dye concentrations at times t and 0, respectively.

Various parameters were studied as Nano photocatalyst MOF: DAC@PdA@FM dose (0.0025–0.01 g), pH (2–8), initial concentration of dye (50–500ppm), contact time (5.0–120 min) and temperature (25.0–45.0 °C). The selected concentrations reflect typical discharge patterns in wastewater, with higher levels of cationic dyes (CV and TBO) compared to E110, enabling a realistic evaluation of competition and selectivity.

### Experimental variables

#### Effect of pH

A dye solution of the initial concentration (200 ppm TBO, 250 ppm CV and 100 ppm E110) was mixed with 0.015 g MOF: DAC@PdA@FM nanocomposite in 25 mL transparent containers. The initial pH was adjusted to specific values (2.0–8.0) using 0.1 M HCl and/or 0.1 M NaOH. The transparent vials were shaken in a balanced shaker at a shaking speed of 150 rpm, in the presence of UV light and at a temperature of 25 °C for 1 h. Typically, the uptake experiments were carried out in this manner. To determine the optimal pH for a MOF pollutant system, it is necessary to take into account different factors. The adsorption equilibrium is affected by pH, which impacts the kinetics.

#### Effect of dose of the MOF: DAC@PdA@FM photocatalyst

To evaluate the impact of the photocatalyst amount, particular masses of **MOF: DAC@PdA@FM** was added to each transparent bottle. Different masses (0.0025, 0.005, 0.0075, and 0.01 gm) were added to transparent bottles containing 25 mL of the dye solution. Then, the photocatalytic procedures achieved at 25 °C for 1 h in the presence of the UV light. The dye’s concentration in the solid phase (Qe) at equilibrium was calculated utilizing (Eq. 4).

#### Effect of initial dye concentration and isotherm studies

0.015 g of MOF: DAC@PdA@FM was mixed with 25 mL of dye solution at varying initial concentrations (50, 100, 150, 200, and 300 ppm). The vials were shaken at 150 rpm under UV light at 25 °C for 1 h. The equilibrium dye concentration in the solid phase (Qe) was determined using a mass balance Eq. 4.

4 $$\:\text{Q}\text{e}=\:\:(\text{C}\text{o}-\text{C}\text{e}\text{q})\:\times\:\frac{V}{m}$$

The parameters related to dye adsorption by a photocatalyst, including the volume of dye solution (v), mass of the photocatalyst (m), uptake value (Qe), and dye concentrations at equilibrium (Ceq) and initial (Co). It mentions the linear forms of the Langmuir and Freundlich isotherm equations, presented in referenced Eqs^5,6^.

5 $$\:\frac{{\text{C}}_{\text{e}}}{{\text{q}}_{\text{e}}}=\frac{1}{{\text{K}}_{\text{l}}{\text{q}}_{\text{m}}}+\frac{{\text{C}}_{\text{e}}}{{\text{q}}_{\text{m}}}$$

6 $$\:\text{ln}{\text{q}}_{\text{e}}=\text{ln}{\text{K}}_{\text{f}}+\frac{\text{ln}{\text{C}}_{\text{e}}}{\text{n}}$$

where C_e_ (ppm) is the dye concentration in the equilibrium state, q_e_ (mg/g) is the capacity of the dye concentration in the equilibrium state, q_m_ (mg/g) is the maximum adsorption value, 1/n, K_L_ and K_F_, Langmuir coefficient (L/mg) and Freundlich constant (mg/g).

#### Effect of the contact time and kinetic studies

Kinetic studies were performed by placing 0.015 g of MOF: DAC@PdA@FM in a series of transparent bottles containing 25 mL (50, 100, 150, 200, and 300 ppm) at ideal pH conditions at 25 °C and shaking time (5–120 min) at 150 rpm in the presence of UV light. In order to illustrate the mechanism of the degradation process and determine the rate of degradation, kinetic studies were carried out using pseudo-first-order (Eq. 7) and pseudo-second-order models (Eq. 8).

7 $$\:\frac{1}{{\text{q}}_{\text{t}}}=\frac{{\text{K}}_{1}}{{\text{q}}_{\text{e}}\text{t}}+\frac{1}{{\text{q}}_{\text{e}}}$$

8 $$\:\frac{\text{t}}{{\text{q}}_{\text{e}}}=\frac{1}{{\text{K}}_{2}{\text{q}}_{\text{e}}^{2}}+\frac{\text{t}}{{\text{q}}_{\text{e}}}$$

The parameters used to characterize degradation efficiency, specifically q_e_ (mg/g) at equilibrium and qt (mg/g) at time t (min). K_1_ and K_2_ as the pseudo-first-order and pseudo-second-order degradation rate constants, respectively. Both models calibrate k and q_e_ together, with the correlation coefficient being utilized to determine the best kinetic model fitting the experimental data.

#### Effect of temperature and thermodynamics studies

A series of transparent bottles containing 25 mL of the solutions (200 ppm TBO, 250ppm CV and 100 ppm E110) and0.015 g of **MOF**: **DAC@PdA@FM**, at optimum pH (6, 8) for cationic dyes and pH (2) for anionic dye, were shaken for1 hours in a balanced shaker at 150 rpm, while temperature was maintained between (25–45) °C in the presence of light source. Residual concentration of dye was determined after photocatalytic degradation. By using (Eq. 9) and (Eq. 10), the thermodynamic parameters as free energy (ΔG^o^), enthalpy (ΔH^o^) and entropy (ΔS^o^) have been calculated.

9 $$\:\varDelta\:{\text{G}}^{\text{o}}=-\text{R}\text{T}\text{ln}{\text{K}}_{\text{c}}$$

10 $$\:\text{ln}{\text{K}}_{\text{c}}=\frac{{|Delta}{\text{S}}^{\text{o}}}{\text{R}}-\frac{{\Delta}{\text{H}}^{\text{o}}}{\text{RT}}$$

As the R (gas constant) equal (8.314 J/mol K), the values of ΔH^o^ were calculated from the slope (−ΔH^o^/R) of ln Kc vs. 1/T, and ΔS^o^ was calculated from the intercept (ΔS^o^/R) of ln Kc vs. 1/T.

#### Error function analysis

To evaluate the goodness-of-fit for the adsorption isotherm and kinetic models, three statistical error functions were applied: $\:\text{C}\text{h}\text{i}-\text{s}\text{q}\text{u}\text{a}\text{r}\text{e}\:\text{E}\text{r}\text{r}\text{o}\text{r}\:\text{F}\text{u}\text{n}\text{c}\text{t}\text{i}\text{o}\text{n}\left({\:\varvec{x}}^{2}\right)$, sum of squared errors (SSE), mean squared error (MSE), hybrid error function (Hybrid) and the relative adsorption error (Δq%). These metrics allow for quantitative comparison between experimental and predicted values:

$$\:\text{C}\text{h}\text{i}-\text{s}\text{q}\text{u}\text{a}\text{r}\text{e}\:\text{E}\text{r}\text{r}\text{o}\text{r}\:\text{F}\text{u}\text{n}\text{c}\text{t}\text{i}\text{o}\text{n}\left({\:\varvec{x}}^{2}\right)$$:

The Chi-square error function (χ²) evaluates the relative difference between experimental and model-calculated values, normalized by the predicted value.

$$\:{\:\varvec{x}}^{2}=\sum\:_{\varvec{i}=1}^{\varvec{n}}\frac{{{(\varvec{q}}_{\varvec{e},\varvec{e}\varvec{x}\varvec{p}}-{\varvec{q}}_{\varvec{e},\varvec{c}\varvec{a}\varvec{l}\varvec{c}})}^{2}}{{\varvec{q}}_{\varvec{e},\varvec{c}\varvec{a}\varvec{l}}}$$

Mean Squared Error (MSE):

MSE normalizes the SSE by the number of data points, allowing comparison across different data sets or models.

$$\:\varvec{M}\varvec{S}\varvec{E}=\frac{1}{\varvec{n}}\sum\:_{\varvec{i}=1}^{\varvec{n}}({{(\varvec{q}}_{\varvec{e},\varvec{e}\varvec{x}\varvec{p}}-{\varvec{q}}_{\varvec{e},\varvec{c}\varvec{a}\varvec{l}\varvec{c}})}^{2}$$

Hybrid Error Function:

The hybrid function combines relative error and model complexity (number of parameters p), providing a more refined assessment of model performance.

$$\:\varvec{H}\varvec{Y}\varvec{B}\varvec{R}\varvec{I}\varvec{D}\varvec{E}=\frac{100}{\varvec{N}-\varvec{P}}\sum\:_{\varvec{i}=1}^{\varvec{n}}\left[\frac{{{(\varvec{q}}_{\varvec{e},\varvec{e}\varvec{x}\varvec{p}}-{\varvec{q}}_{\varvec{e},\varvec{c}\varvec{a}\varvec{l}\varvec{c}})}^{2}}{{\varvec{q}}_{\varvec{e},\varvec{c}\varvec{a}\varvec{l}}}\right]$$

Sum of Squared Errors (SSE):

SSE reflects the cumulative deviation between observed and predicted adsorption capacities.

$$\:SSE=\sum\:_{i=1}^{n}{{(q}_{e,exp}-{q}_{e,calc})}^{2}$$

The relative adsorption error (Δq%) was calculated to assess the percentage deviation of the model predictions from the experimental results, using the expression:

$$\:\varDelta\:\varvec{q}\varvec{\%}=100\sqrt{\frac{\sum\:\left({\frac{{\varvec{q}}_{\varvec{e},\varvec{e}\varvec{x}\varvec{p}}-{\varvec{q}}_{\varvec{e},\varvec{c}\varvec{a}\varvec{l}\varvec{c}}}{{\varvec{q}}_{\varvec{e},\varvec{e}\varvec{x}\varvec{p}}}}^{2}\right)}{\varvec{n}-1}}$$

where:

qe_exp_: experimentally observed equilibrium adsorption (mg/g).

qe_calc_: predicted value from the model.

n: number of observations.

p: number of model parameters.

These statistical measures were used to support the selection isotherm and kinetic models in Sect. “Photocatalytic studies”.

### **The reusability of MOF: DAC@PdA@FM nanocomposite photocatalyst**

The DAC@PdA@FM composite was regenerated through five cycles of adsorption and desorption. Degradation experiments were conducted using 0.005 g of the composite and 25 mL of TBO, CV, and E110 solutions at concentrations of 200, 250, and 100 mg/L at pH 6.0, 8.0, and 2.0, respectively, for 30 min. For desorption, 25 mL of Ethanol, 0.1 M NaOH and Na_2_CO_3_ were used with 0.005 g of DAC@PdA@FM, shaken for 30 min. The regenerated composite was then recycled for four more cycles of adsorption and desorption process.

### Application

In the spiking process, TBO, CV, and E110 dyes were added to tap, sea, and wastewater samples at concentrations of 200, 250, and 100 mg/L, respectively. Before adding the dyes, the water samples were treated with a mixture of K_2_S_2_O_8_ (0.5 g) and H_2_SO_4_ (5 mL, 98% w/w) and heated at 90 °C for 120 min to digest organic materials^58^. After cooling, 0.005 g of MOF:DAC@PdA@FM nanocomposite was added to each sample, and the pH was adjusted to 6.0, 8.0, and 2.0 for TBO, CV, and E110 dyes, respectively. The samples were then shaken for 30 min, centrifuged, and another 0.005 g of DAC@PdA@FM was added to the supernatant to ensure complete separation of the dyes^58^. The remaining amounts of TBO, CV, and E110 were determined spectrophotometrically at appropriate wavelength.

### Computational investigation methods

Geometry optimizations and other DFT calculations were performed on the cellulose unit, monosodium cellulose, DAC, DAC@PdA, and DAC@PdA@FM nanoparticles composite. DFT is considered a cost-effective method to approximate electron correlation effects. All DFT calculations were performed by using B3LYP level of theory, Becke’s three parameter (B3) nonlocal exchange with the correlation functional of Lee, Yang, and Parr (LYP)^59^. The B3LYP functional was selected for its proven accuracy in predicting geometries and energy gaps in organic and organometallic systems. The 6–311 + + G(d, p) basis set provides sufficient flexibility for accurate electronic property calculations of the MOF system. Nowadays, the B3LYP level is currently widely used to study organic electronic compounds because the predicted geometries are very reliably and provides good estimations for HOMO–LUMO gaps, in a good agreement with experimental values^60–63^. All the calculations, for geometry optimizations were carried out at the B3LYP/6–31 g (d, p) for all atoms. All computations were carried out by using Gaussian09 suite of program^64^. IR spectrum were graphed using GaussSum2.2.5 program^65^and full natural bond orbital (NBO) analyses were made to calculate the charge distribution for all molecules by using NBO version 3.1^66^. Gauss View 5.0 package^67^ was used to obtain various graphic views of molecular shapes of distinctive molecular orbitals.

Highest Occupied Molecular Orbitals (HOMO) and Lowest Unoccupied Molecular Orbitals (LUMO) are very considerable elements of theoretical molecular design^68^. The electronic properties and reactivity definers [such as ionization potential (I_P_), electron affinity (E_A_), hardness ($\:\eta\:)$, softness ($\:\sigma\:$), electronegativity ($\:\chi\:$)] can be determined from the HOMO and LUMO orbital energies through Koopman’s theorem^69^.

The Global Reactivity Parameters; Ionization potential (I_P_), electron affinity (E_A_), the electronegativity ($\:\chi\:$), global hardness ($\:\eta\:)$ and softness ($\:\sigma\:$), can be explained in terms of the energy of the HOMO and the LUMO in Table 2^70–74^.

**Table 2:** Global reactivity descriptors (GRD).

GRD	Equation
**Ionization potential (I**_**P**_ **)**	I_P_ = - E_HOMO_
**Electron affinity (E**_**A**_ **)**	E_A_ = - E_LUMO_
**The energy gap (ΔE**_**gap**_ **)**	ΔE_gap_ = (E_LUMO_ - E_HOMO_)
**Hardness (η)**	$\:\eta\:=\frac{{I}_{P}-\:{E}_{A}}{2}$
**Softness (** $\:\varvec{\sigma\:}$ **)**	$\:\sigma\:=\frac{1}{\eta\:}$
**Electronegativity (χ)**	$\:\chi\:=\frac{{I}_{P\:}+{\:E}_{A}}{2}$
**Electrophilicity index (ω)**	(ω) = $\:\frac{\:{{\upmu\:}}^{2}}{2\eta\:}$
**Electronic chemical potential**^75^ $\:\left(\varvec{\mu\:}\right)$	$\:\left(\mu\:\right)=-\:\text{E}\text{l}\text{e}\text{c}\text{t}\text{r}\text{o}\text{n}\text{e}\text{g}\text{a}\text{t}\text{i}\text{v}\text{i}\text{t}\text{y}=\:\mu\:=\:-\:{\upchi\:}$
**The Global electrophilicity index** ($\omega$)	$\:\left(\:{\omega}\:\right)=\:\frac{{\mu\:}^{2}}{2{\upeta\:}}$

### Antimicrobial activity studies

The antimicrobial activities of the MOF: DAC@PdA@FM nanocomposite were tested in the microbiology lab at the Center of Genetic Engineering and Biotechnology, Mansoura University, using the agar well diffusion method. The study included positive control (Azithromycin) as antibiotic and both gram-positive (S. aureus, Bacillus subtilis, Enterococcus colaceae) and gram-negative (E. coli, Klebsiella pneumonia) bacteria. Microbial inoculum was spread over agar plates, and 9 mm holes were punched for the test substance. About 50 µL of dissolved MOF: DAC@PdA@FM nanocomposite was added to the plates, and after incubation, the inhibition zones were measured in millimeters^76^.

## Results and discussion

### Materials’ design

The digital photographs of native cellulose, DAC, DAC@PdA, Fe_3_O_4_, and DAC@PdA@FM illustrate a clear color transformation during modifications as it is shown in Fig. 1S(a-c). The DAC initially appears white, turning reddish-brown after PdA modification and brown after Fe_3_O_4_ modification. Subsequent dye adsorption further altered the color of DAC@PdA@FM, indicating its effective dye adsorption capability.

DAC@PdA@FM is a brown crystalline material with high thermal stability. It withstands temperature above 400^o^C and it is partially soluble in DMSO and acidic media (conc. HCl). The use of the metal-organic framework nanocomposite (DAC@PdA@FM) aimed at designing optimum material that interact well with cationic and anionic dyes guaranteeing optimal performance under suitable conditions. The incorporation of magnetic nanoparticles (Fe_3_O_4_) was used to separate the composite from aqueous solutions by applying an external magnetic field. Characterization of the composite was performed to confirm its composition, performance and magnetic properties. (FTIR) was applied to confirm the presence of functional groups responsible for dye degradation. (XRD) and (SEM) were used to ensure the crystallinity and structure of the nano MOF composite and to notice the morphology and distribution of the nano composite components. After that, the composite’s photocatalytic degradation capacity was assisted for specific cationic and anionic dyes under varied methods (pH, temperature, time, dye concentration). Kinetic studies analyze the degradation rates of dyes using pseudo-first and second-order pseudo-models. Isothermal models, including Langmuir and Freundlich, evaluate photocatalytic degradation process and energies. In addition, usability and stability tests evaluate the performance of the composite in different adsorption-desorption cycles for usability.**DAC synthesis** involves the use of the oxidizing agent KIO_4_, which selectively oxidizes the hydroxyl groups on adjacent carbon atoms C_2_-C_3_ of the glucopyranoside ring, converting them into dialdehyde groups. The oxidation degree measures the percentage of monosaccharide units reacted with KIO_4_ to determine aldehyde content. As well, The Mwt of the DAC repeating unit (C_6_H_8_O_10_)*n* = 160.124 g/mol.The AC percentage of synthesized DAC is 39.8% shown in Table S1.

### Characterization

#### Elemental analysis

Table 3 presents the elemental analysis results for native cellulose^77^ and modified cellulose (DAC@PdA). Native cellulose has a carbon content of 44.45%, while DAC@PdA shows an increase to 46.23% due to the addition of phenylenediamine. Although native cellulose has a higher hydrogen content (7.24%) compared to modified cellulose (5.87%), this decrease is likely a result of aldehyde group formation during the oxidation process. Notably, nitrogen content increased significantly to 7.7399% in the modified cellulose, indicating successful modification through the introduction of phenylenediamine’s amine groups.

**Table 3:** Elemental analysis of native cellulose and the prepared DAC@PdA.

Material	C%	H%	*N*%
Native cellulose	44.45	7.24	0
DAC@PdA	46.2339	5.8745	7.7399

#### SEM and EDS

**Fig. 2:**
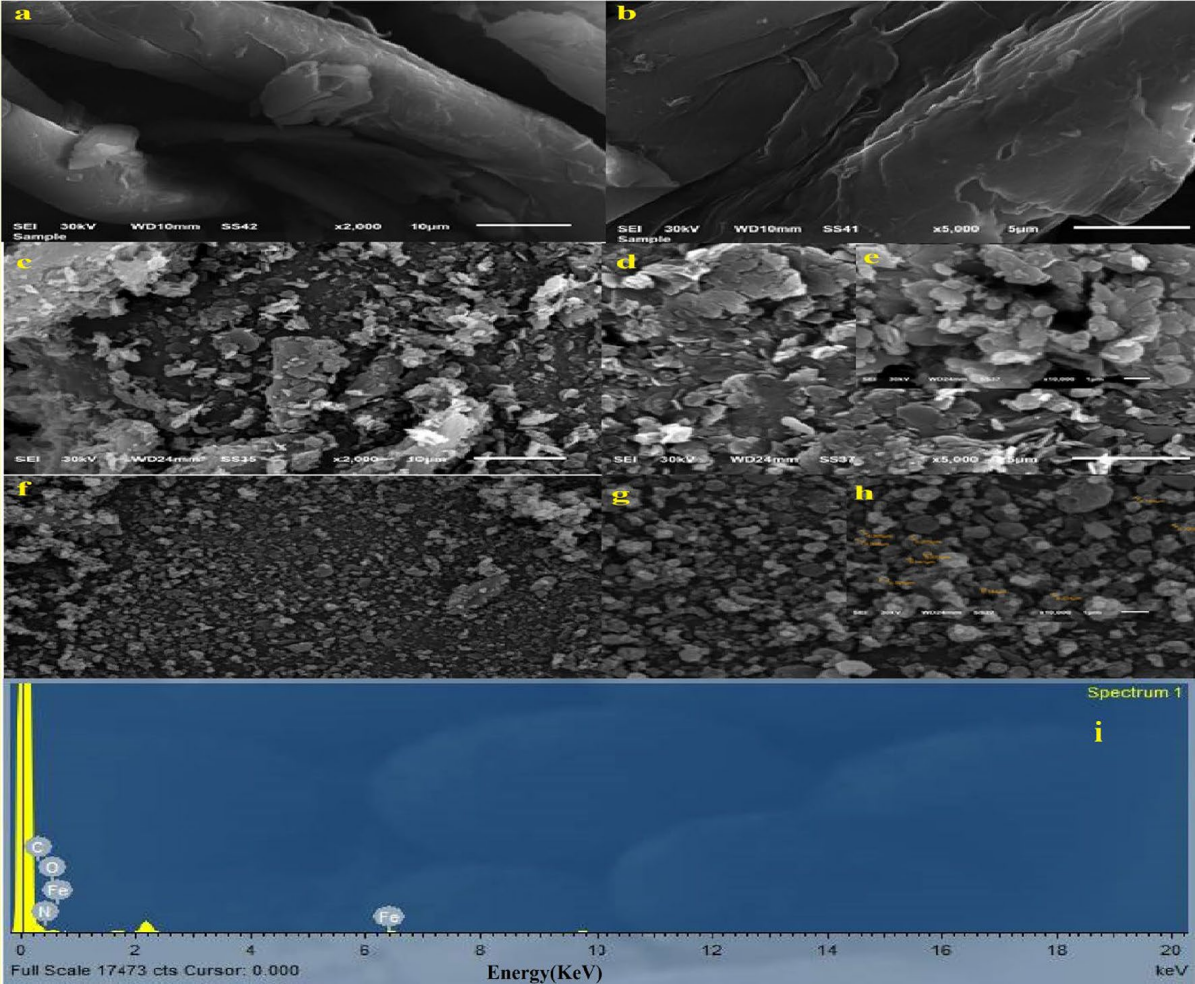
(a, b) SEM images of oxidized cellulose (DAC) at magnifications of 2000× and 5000×, respectively.(c, d, e) SEM images of DAC@PdA composite at various magnifications showing the surface modification.(f, g, h) SEM images of MOF:DAC@PdA@FM nanocomposite showing the distribution of Fe₃O₄ particles.(i) EDX spectrum of MOF:DAC@PdA@FM nanocomposite. The x-axis represents energy (keV) and the y-axis represents counts (intensity of detected X-rays).

The SEM analysis of DAC, DAC@PdA, and the MOF:DAC@PdA@FM nanocomposite at various magnifications (10 μm, 5 μm for DAC, DAC@PdA, and DAC@PdA@FM and 1 μm for DAC@PdA, and DAC@PdA@FM) reveals distinct structural characteristics. As shown in Fig. 2(a-h)., DAC displays a smooth structure, indicating an unmodified cellulose matrix. In contrast, DAC@PdA presents increased roughness, highlighting successful modification with phenylenediamine (PdA), which may enhance surface properties. As seen, In the DAC@PdA@FM sample at 1 μm scale, the presence of nanoparticles is highly likely The MOF:DAC@PdA@FM nanocomposite exhibits the most irregular morphology due to the addition of Fe_3_O_4_ nanoparticles, resulting in larger particle aggregation and potentially higher surface area, improving adsorption and catalytic activity. The presence of these nanoparticles suggests enhanced interaction capabilities, confirming the effectiveness of the modification process of DAC with PdA and Fe_3_O_4_^78,79^. As well, EDS analysis confirms the presence of main elements (C, O, N, Fe) corresponding to modified cellulose (DAC@PdA) and Fe_3_O_4_ composite. The integration of Fe_3_O_4_ with (DAC@PdA) structure is successful, as shown by the strong iron signals, while the components of (carbon, oxygen, nitrogen) are found in the spectrum, which indicates (DAC@PdA) is well presented in the Final composite of (DAC@PdA@FM), as seen in Fig. (2i)^80^.

#### FTIR analysis

**Fig. 3:**
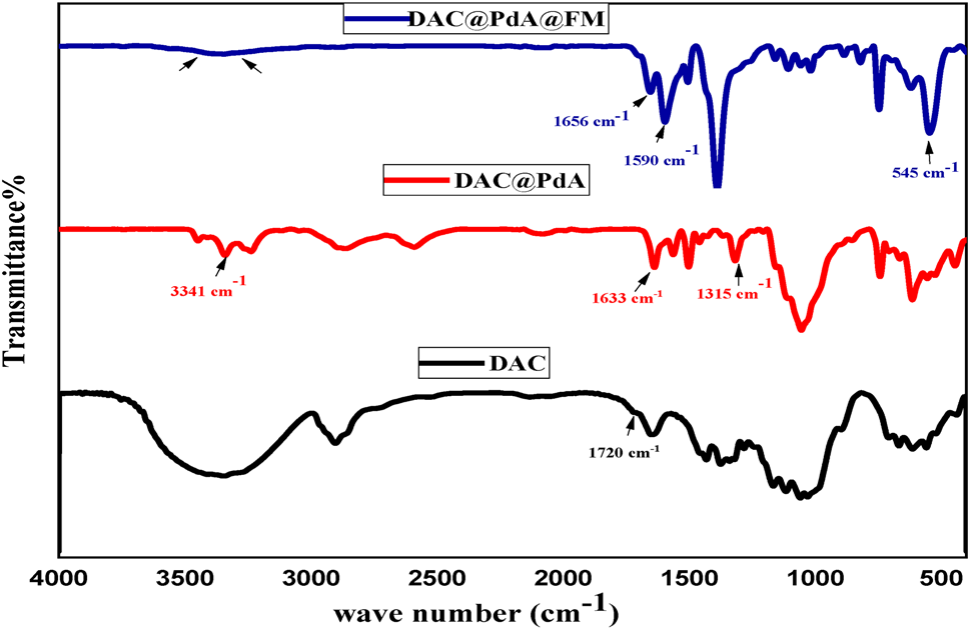
FTIR spectra of DAC, DAC@PdA, and MOF: DAC@PdA@FM nanocomposite.

FTIR spectra of DAC, FTIR spectra of DAC, DAC@PdA, and the MOF:DAC@PdA@FM nanocomposite confirmed the successful modification of cellulose through distinct changes in functional groups as shown in Fig. 3. The dialdehyde cellulose (DAC) synthesis was proved by the characteristic absorption band of carbonyl vibrational frequency at 1720 cm^−1^ in the spectrum. Additionally, the peaks at 3390, 2912, and 1647 were ascribed to OH stretching, CH_2_ stretching, and OH bending respectively. The peaks at 1372 and 1172 cm^−1^ were attributed to CH_2_ bending and C–O–C stretching vibrations. In the FT-IR spectrum of DAC@PdA one NH_2_ group of PdA was found to bind with a carbon atom of DAC aldehyde group through C-N bond as identified at 1315 cm^−1^. Moreover, the observed peak at 1633 cm^−1^ is belonging to the aromatic (C = C) of PdA and a peak at 1004 cm^−1^ is corresponding to (C-C). The detected peak at 3341 cm^−1^ is related to N = H in secondary amine of PdA. The FT-IR spectrum of DAC@PdA@FM nanocomposite shows a characteristic band at 545 cm^−1^, which confirms Fe–O bonds in the crystal structure of Fe_3_O_4_^81^. The other characteristic bands were observed between 1590 and 1656 cm^−1^ related to N–H and C-N groups. The characteristic bands of symmetrical –CH group and aromatic –C–H bond of benzoic acid was at 1390, and (644–728) cm^−1^. Also, the characteristic bands at 832 and 888 cm^−1^ resulting from the –CH and-OH of Terephthalic acid confirming the synthesis of MOf^82^. The obvious decreasing of the-OH and -NH bands intensity for DAC@PdA@FM nanocomposite was ascribed to the forming of amide carbonyl group, indicating that many Fe_3_O_4_ nanoparticles have been covalently immobilized on DAC@PdA component with terephethalic acid in the DAC@PdA@FM. Moreover, it was noticeable that there were a lot of different types of sorption sites such as carboxyl group, amide group, carbonyl group, amino group, and hydroxyl group on the DAC@PdA@FM composite, which could contribute to the sorption of not only cationic but also anionic dye pollutants, plus other sorts of pollutant.

#### **FTIR of MOF:DAC@PdA@FM nanocomposite after dyes’ degradation**

**Fig. 4:**
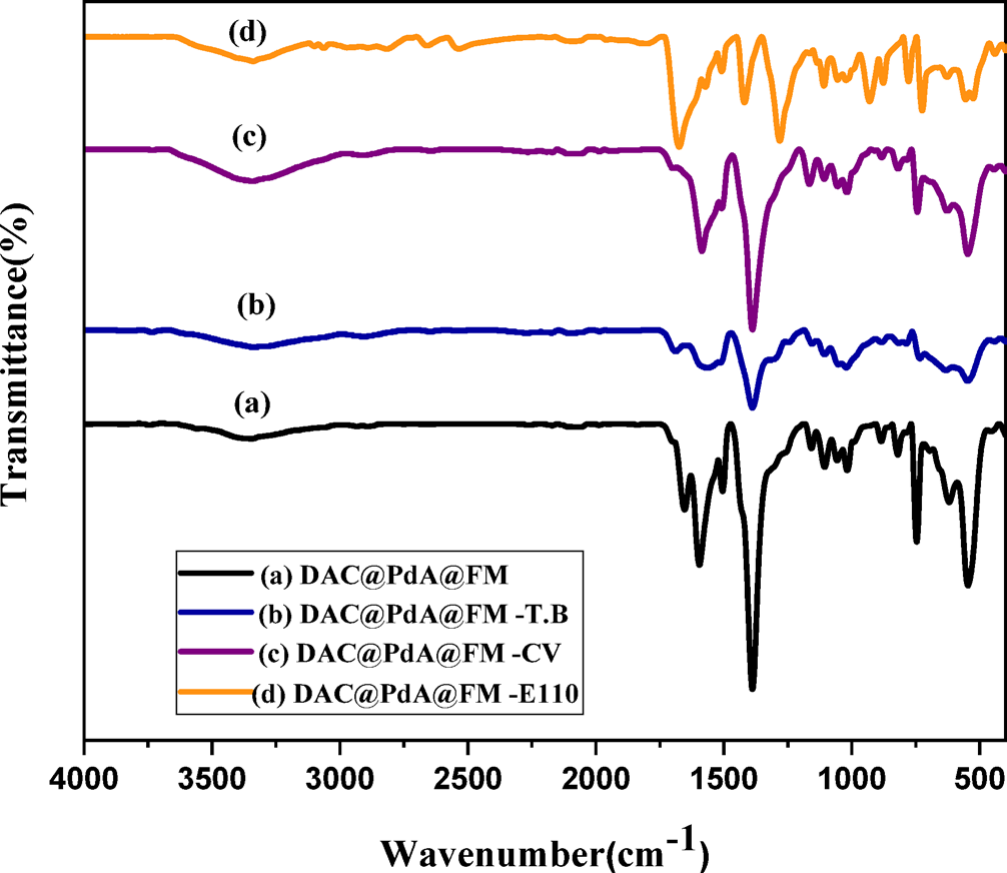
FTIR of MOF:DAC@PdA@FMnanocomposite with dyes’ degradation.

From the FTIR spectrum (Fig. 4.), we can interpret the type of interactions between the MOF:**DAC@PdA@FM** nanocomposite and the dyes ( CV, TBO, and E110), through analyzing the changes in intensities, shifts in peaks, and the appearance of new peaks. There is an intensity change appearing in the region around 3500–3200 cm⁻¹ (O-H or N-H stretching) for CV and E110^31,83^. This indicates the formation of hydrogen bonds with hydroxyl groups (-OH) in DAC or with the amine groups (-NH_2_) in MOF: **DAC@PdA@FM nano** composite. There is a shift in the C = O stretching in the 1700–1600 cm⁻¹ range for both CV and TBO are cationic (positively charged)^84^. So, Electrostatic attractions between the positive dye molecules and the negative sites in the MOF:**DAC@PdA@FM** (carboxyl groups) might occur. There are shifts in the aromatic C = C stretching region around 1600–1500 cm⁻¹. As both CV and TBO have aromatic rings, which can interact with aromatic structures in the **DAC@PdA@FM** through π-π stacking interactions. So, FTIR spectra confirmed the successful modification through the new peaks (Fe–O, amide) and the shifts in O–H/N–H bands, indicating structural integration and improved dye interaction capacity.

#### Thermal analysis (TGA)

**Fig. 5:**
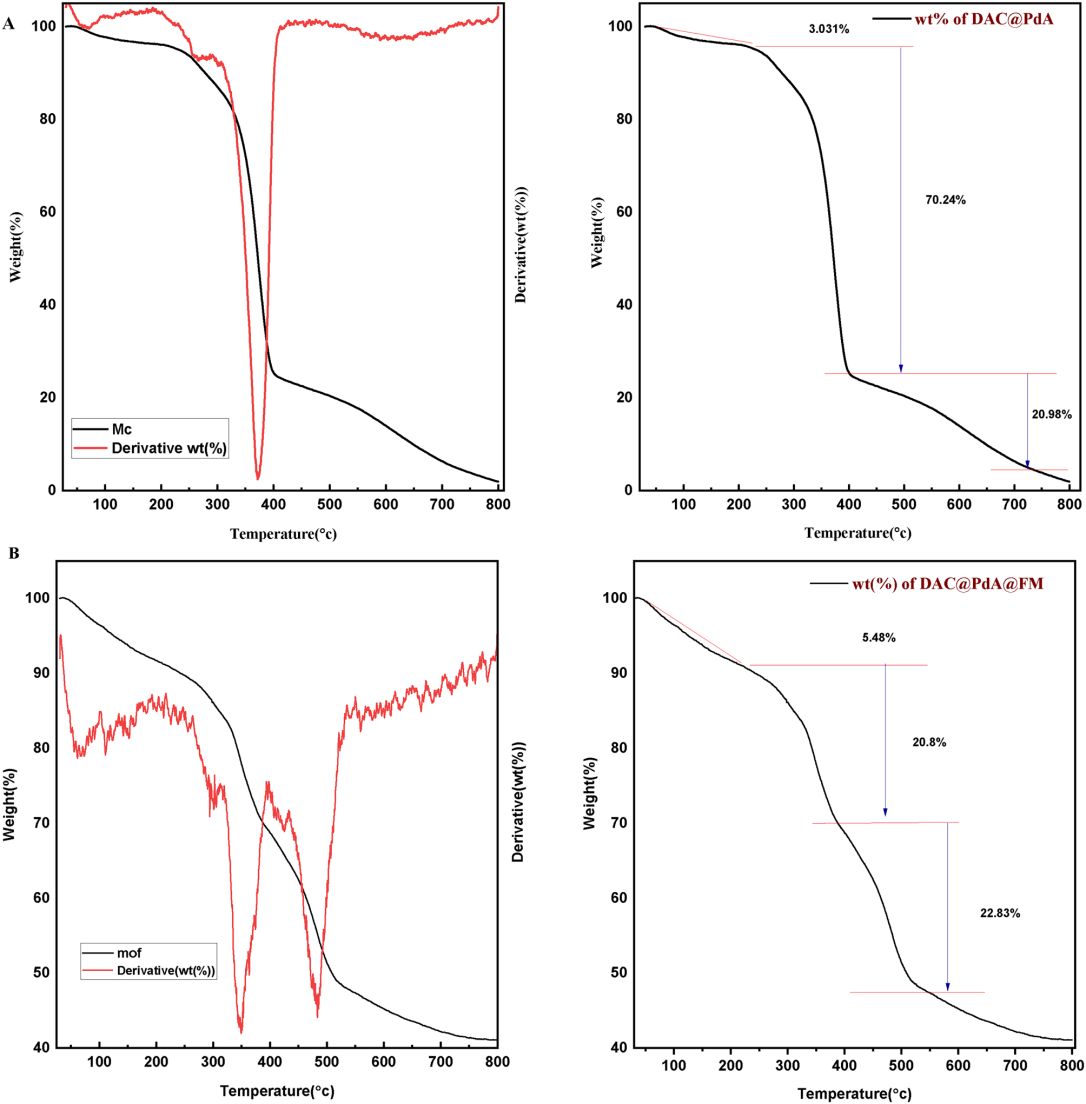
Thermal analysis of (A) DAC@PdA and (B)MOF:DAC@PdA@FM nanocomposite.

The TGA results, Fig. 5a, indicate that DAC@PdA shows a typical organic material decomposition pattern with an inorganic phase. The initial weight loss of ~ 3.03% corresponds to moisture, while the main decomposition (~ 71.24%) involves the organic substances. The final residue (~ 20.38%) indicates non-combustible materials like iron oxide. For the DAC@PdA@FM composite (Fig. 5b), a three-step degradation process is observed: initial weight loss (~ 5.43%) from water or volatiles, major loss (~ 51.8%) from organic linkers decomposition, and residual weight (~ 22.83%) from inorganic metal oxide components after heating^85^.

#### XRD analysis

**Fig. 6:**
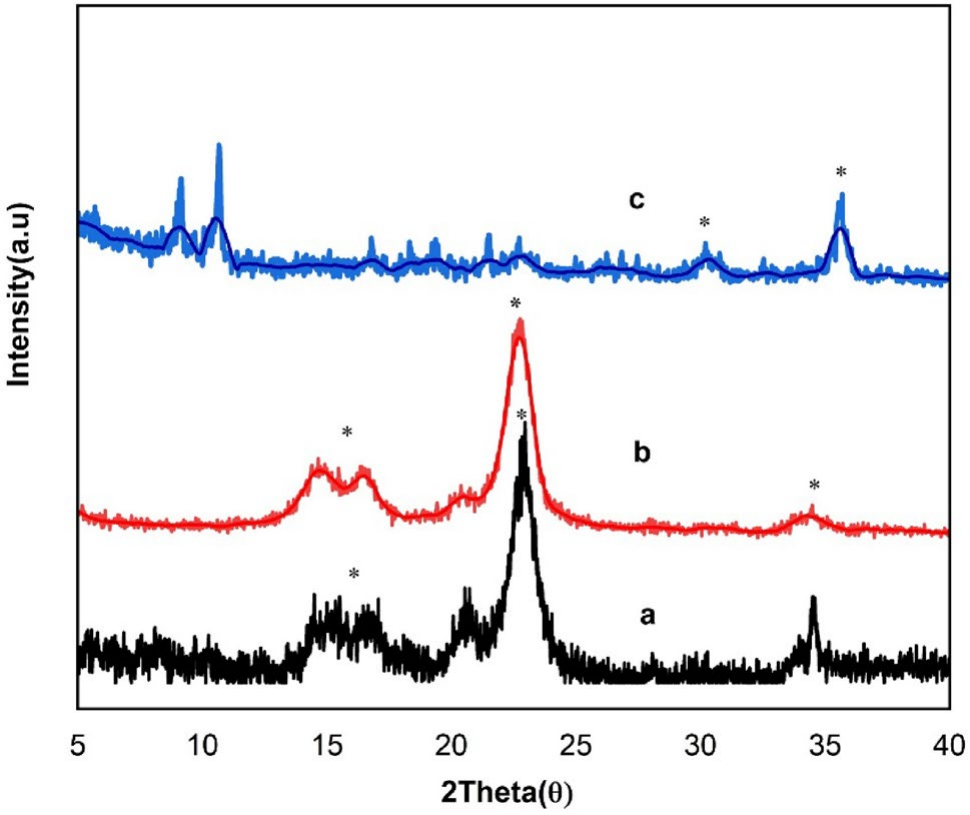
XRD patterns of (**a**) DAC, (**b**) DAC@PdA, and (**c**) MOF:DAC@PdA@FM nanocomposite.

From Fig. 6, the X-ray diffraction patterns provided further insights into the crystalline nature of the composite. The native cellulose (DAC) exhibited a semi-crystalline structure with broad peaks around 15–22° (2θ)^77^. Modification with PdA resulted in a slight reduction in crystallinity, as evident from peak broadening. The incorporation of Fe₃O₄ introduced sharp peaks at 30°, 35°, 43°, and 57° (2θ), characteristic of magnetite nanoparticles. These results confirm the successful formation of a hybrid magnetic nanocomposite with a well-defined structure. The presence of Fe₃O₄ slightly reduced whole crystallinity, as shown by peak broadening, which indicates increased surface area and defect sites that enhance photocatalytic activity^86^.

#### UV/Vis

##### The UV-Vis absorption spectrum of Fe_3_O_4_

**Fig. 7:**
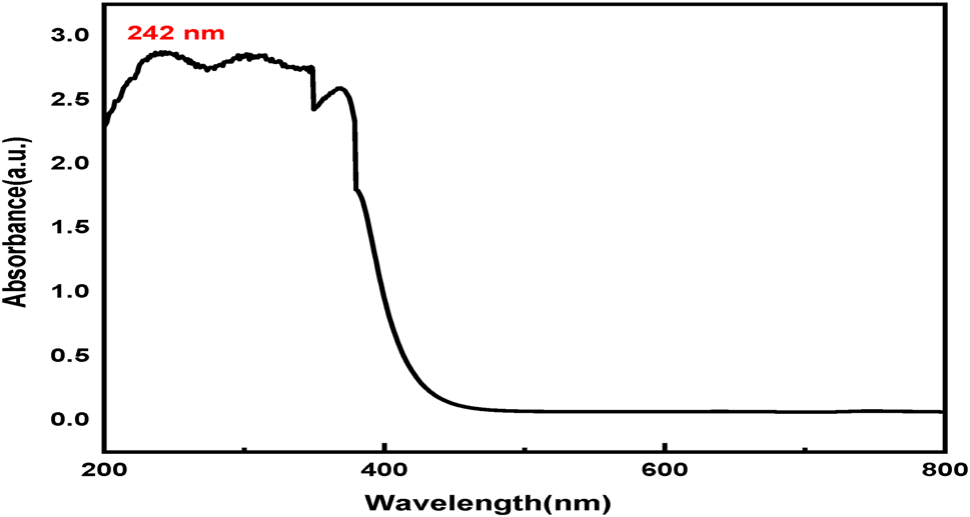
The UV-Vis absorption spectrum of Fe_3_O_4_.

The UV-Vis absorption spectrum of Fe_3_O_4_ magnetite (Fig. 7) shows the absorption as a function of wavelength, with the characteristic peak at 242 nm, showing charge transfer related to Fe-O bonds. This peak could potentially exhibit the LMCT band, which is characteristic of Fe_3_O_4_ nanoparticles. It shows strong absorption in the UV region, which corresponds to the black appearance of Fe_3_O_4_ due to its broad spectrum^87^.

##### The UV-Vis absorption spectrum of single and multicomponent systems

**Fig. 8:**
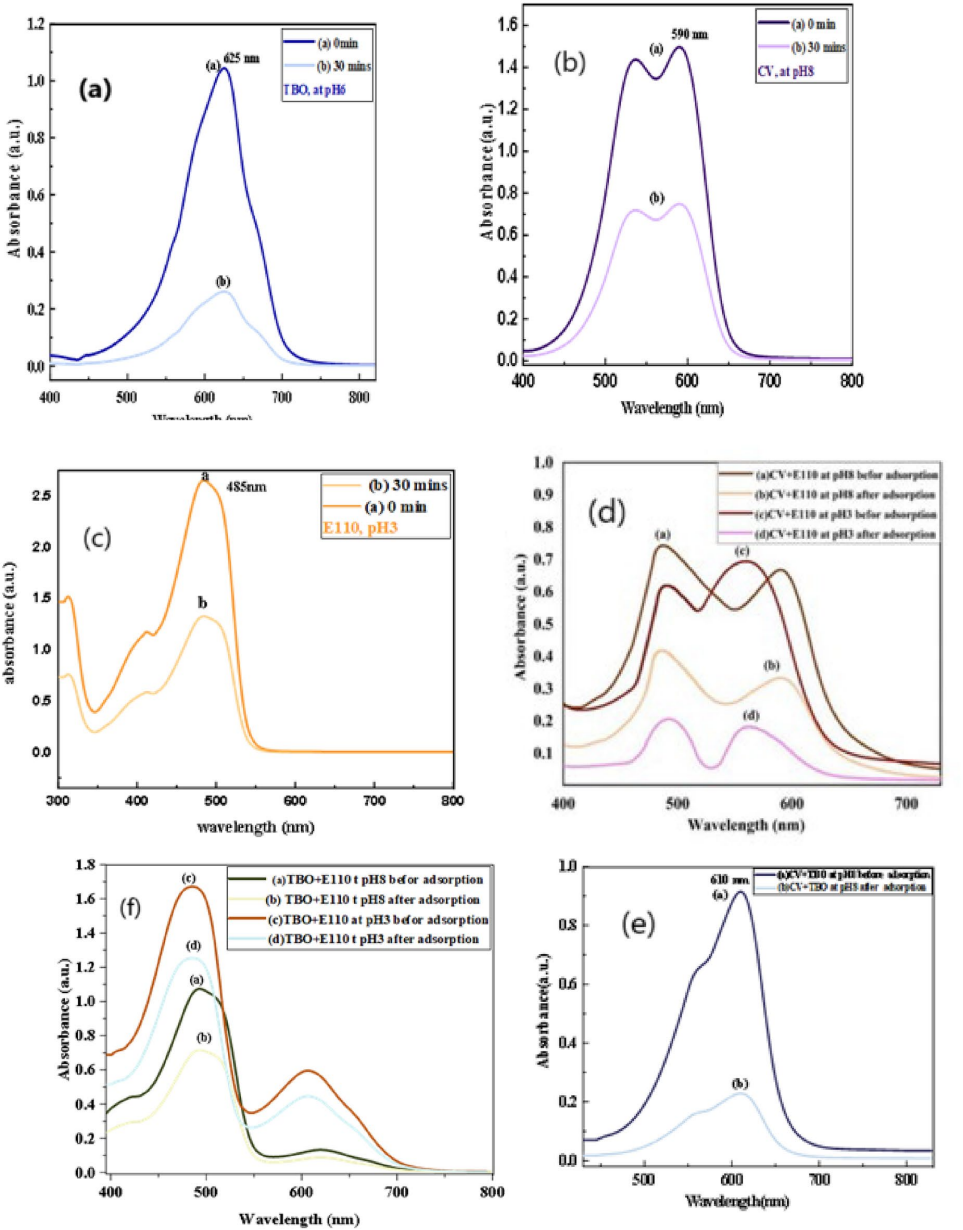
UV-Vis absorption spectra of: **(a**) TBO, (**b**) CV, (**c**) E110, (**d**-**f**) mixture of TBO, CV, E110 after degradation by MOF:DAC@PdA@FM nanocomposite.

The maximum absorbance wavelengths (λ max) for the dyes CV (590 nm), TBO (625 nm), and E110 (485 nm) were assessed in (Fig. 8), and the degradation of these dyes using the MOF: DAC@PdA@FM nanocomposite as a photocatalyst was evaluated. The absorbance peaks of the dyes decreased over time, indicating effective degradation by the photocatalyst. In mixed dye experiments (200 ppm TBO, 250 ppm CV, 100 ppm E110), peak shifting and overlapping were noted. Initially, a broad absorption band suggested the presence of chromophores, while after 30 min of photocatalysis, a significant decline in absorbance indicated effective degradation caused by reactive oxygen species generated under light, confirming the photocatalytic process’s efficiency in reducing color intensity and breaking down chromophores as shown in the Fig. 8(a-c). The study observed, as in Fig. 8(d-f), the formation of new overlapping peaks with distinct λmax values after mixing dyes (610 nm at pH 8.0 for CV-TBO, 560,485 nm at pH 3.0 for CV-E110, 590,480 nm at pH 8.0 for CV-E110, and 606, 485 nm at pH 3.0 for TBO-E110 nm, and 620, 493 nm at pH 8.0 for TBO-E110), specifically at various pH levels for different dye combinations. For the photocatalytic degradation experiment, a catalyst (DAC@PdA@FM) was tested with dye mixtures at concentrations of 100 ppm E110, 200 ppm TBO, and 250 ppm CV, under varying pH conditions. Results indicated that the photocatalyst effectively removes TBO, E110, and CV from binary mixtures, confirming its efficiency for both dyes without significant interference. UV-Vis analysis showed reduced peak intensities, confirming successful dye adsorption^88,89^. In the UV-Vis spectra of the multicomponent dye system (CV, TBO, and E110), detectible shifts in absorption maxima were observed upon treatment with DAC@PdA@FM. These shifts indicate changes in the electronic environments of the dyes, which suggest strong interactions between the dye molecules and the active sites on the composite surface. Forethere, the difference in the dyes shifts propose differential adsorption modes due to the π–π stacking and electrostatic interactions. These spectral changes also support a stepwise degradation mechanism, where the dyes do not degrade simultaneously but follow competitive and selective adsorption and photocatalytic breakdown. The progressive decrease in peak intensity, alongside the shifts, exposes the formation of intermediate species, confirming that the MOF:DAC@PdA@FM nanocomposite facilitates not only dye removal but also structural transformation through catalytic oxidation.

#### Magnetic property and magnetic separation performance

**Fig. 9:**
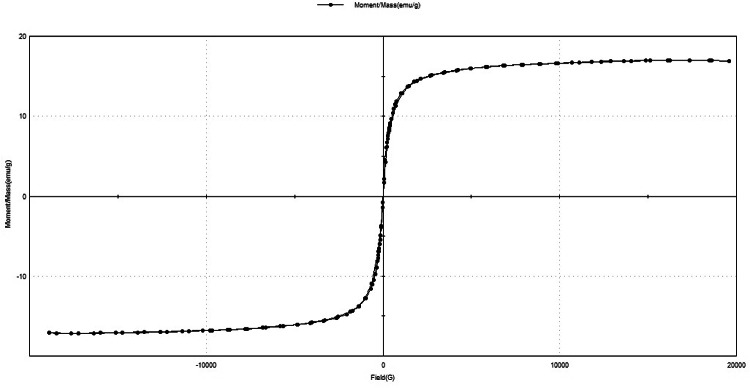
Hysteresis loop of MOF:DAC@PdA@FM nanocomposite.

In order to study these critical properties of prepared magnetic MOF we used PPMS at room temperature; the hysteresis loop in the magnetization curve presented in **Fig.** (9) Illustrates that, the remanence (residue magnetization) and coercive force (the applied field that reduces magnetization to zero) were zero and there was no magnetic hysteresis loop observed, indicating the characteristic superparamagnetic behavior of the prepared MNPs. This is expected for magnetic materials with size less than 10 nm^90^. This result also indicates that only single domain MNP is present in MNPs samples. Superparamagnetic (i.e. responsiveness to an applied magnetic field without retaining any magnetism after removal of the magnetic field) is an especial important property needed for magnetic targeted carriers in drug delivery, as they do not retain magnetization before and after exposure to an external magnetic field, reducing the probability of particle aggregation in the smallest capillaries due to their magnetic dipole attraction. The saturation magnetization (Ms) value read from the magnetization plot in **Fig. (8)** was found to be 16.8 emu/g for MOF. This saturation magnetization of MOF is lower than that of pure Fe_3_O_4_, which may be attributed to the impact of macromolecules and carbon-based materials in composites. Generally, nanoparticles with saturation magnetizations of 16.3 emu/g could be separated magnetically from solution using a magnet. Thus, the MOF:DAC@PdA@FM nanocomposite prepared in this study can be- easily- separated from aqueous solutions, and can -possibly- be ideal adsorbents and carrier. The MOF:DAC@PdA@FM can achieve high-efficiency separation through an external magnetic field after selective adsorption-photocatalytic degradation of investigated dyes.

**Fig. 10:**
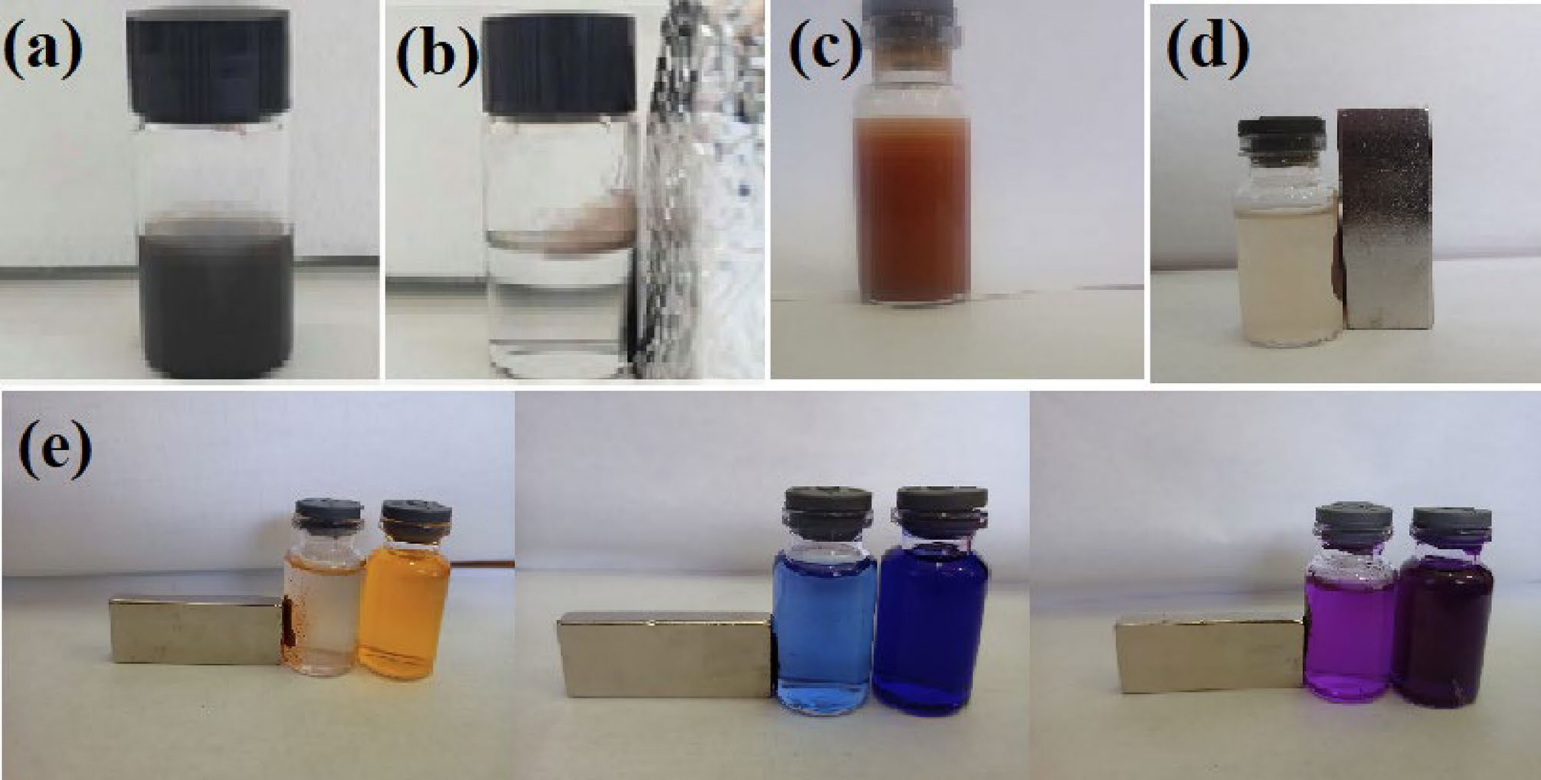
The digital photographs of (a)dispersed Fe_3_O_4_ nano particles, (b) Fe_3_O_4_ nano particles collected with external magnet, (c) MOF:DAC@PdA@FM nanoparticles composite dispersed, (d) MOF:DAC@PdA@FM nanoparticles composite collected with external magnet and (e) MOF:DAC@PdA@FMnano composite with adsorbed and collected dyes(TBO, CV and E110).

To study the magnetic separation effect of MOF: DAC@PdA@FM nanocomposite after adsorption-photocatalytic degradation of dyes, a magnetic field was gradually applied to MOF: DAC@PdA@FM saturated with adsorbed dyes. The comparison results are shown in Fig. 10(a-e). After MOF: DAC@PdA@FM adsorbs dyes, they are evenly dispersed in the solution to form a suspended turbid liquid when the magnetic field is not applied. However, the magnetic field gradually approaches, the MOF:DAC@PDA@FM with adsorbed dyes migrates in the direction of the magnetic field and finally adheres to the wall, and the solution becomes clear. After the external magnetic field moves away, the separated DAC@PdA@FM with adsorbed dyes and the clarified liquid will become turbid again. These results show that by applying an external magnetic field, DAC@PdA@FM after the adsorption of dyes can be separated quickly from the solution to recycle dye resources. Furthermore, the redispersion of DAC@PdA@FM with adsorbed dyes in the solution after removal of the external magnet indicates that these particles loose their magnetisation immediately after removal of the external magnet.

The digital photographs of native cellulose, oxidized cellulose (DAC), and PdA-modified cellulose (DAC@PdA) are presented in Fig.S1, showing the progression of color from white (native cellulose) to reddish-brown (after PdA modification). In contrast, the images of Fe₃O₄ nanoparticles and the MOF:DAC@PdA@FM nanocomposite are shown in Fig. 10, where further darkening to brown indicates successful incorporation of magnetic nanoparticles. Additionally, dye adsorption alters the color of the MOF:DAC@PdA@FM nanocomposite, reflecting its effective adsorption capacity.

### DFT studies

#### Optimized geometry

**Fig. 11:**
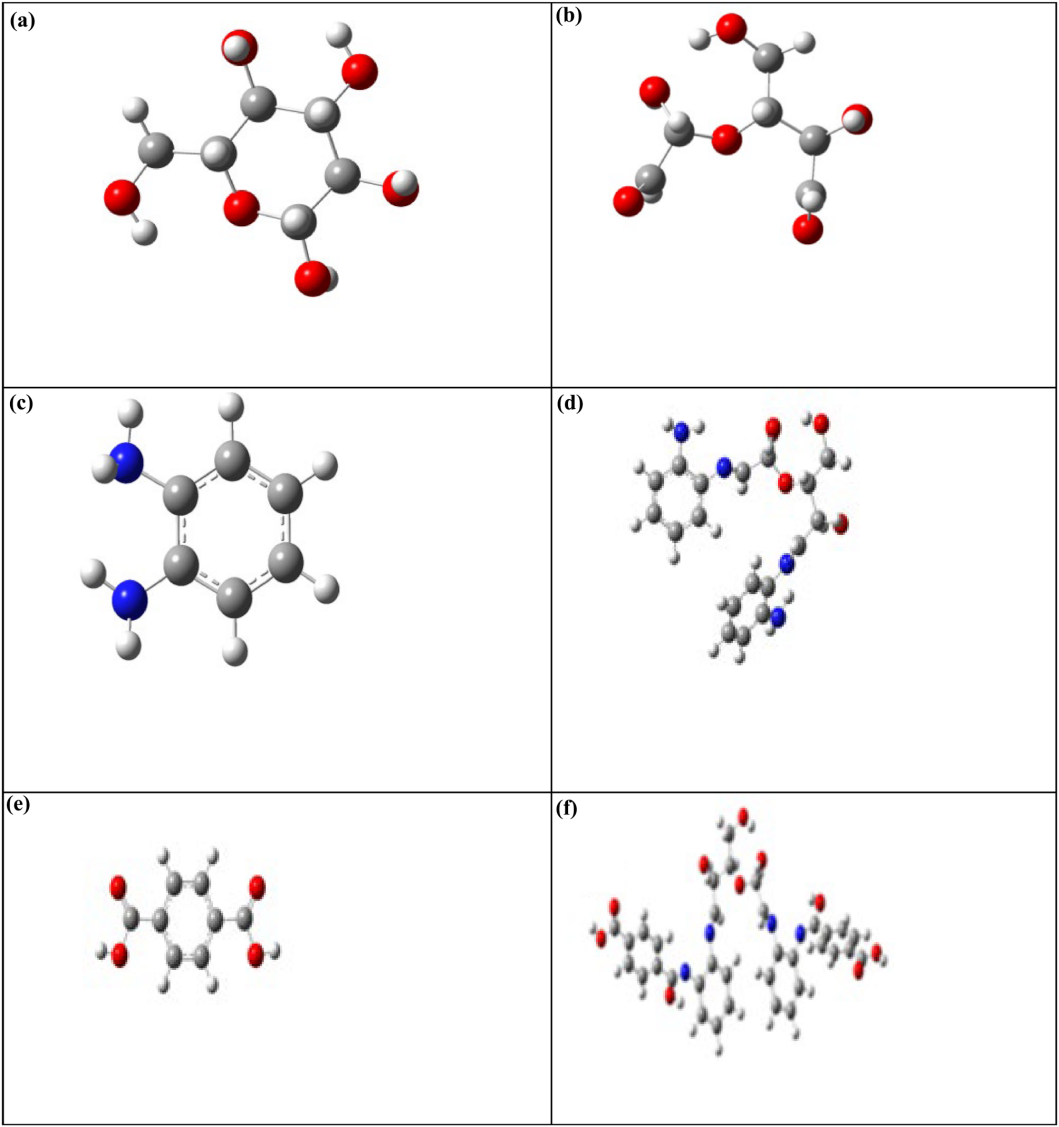
The optimized structures for (**a**) cellulose unit, (**b**) DAC, (**c**) PdA, (**d**) DAC@PdA, (**e**) TPA and (**f**) DAC@PdA@TPA based on the DFT/B3LYP/6–31 g(d, p) methodology.

The optimized geometry of the cellulose unit, DAC, PdA, DAC@PdA, TPA, and DAC@PdA@TPA are shown in Fig. (11).

#### **The global reactivity parameters**

**Fig. 12:**
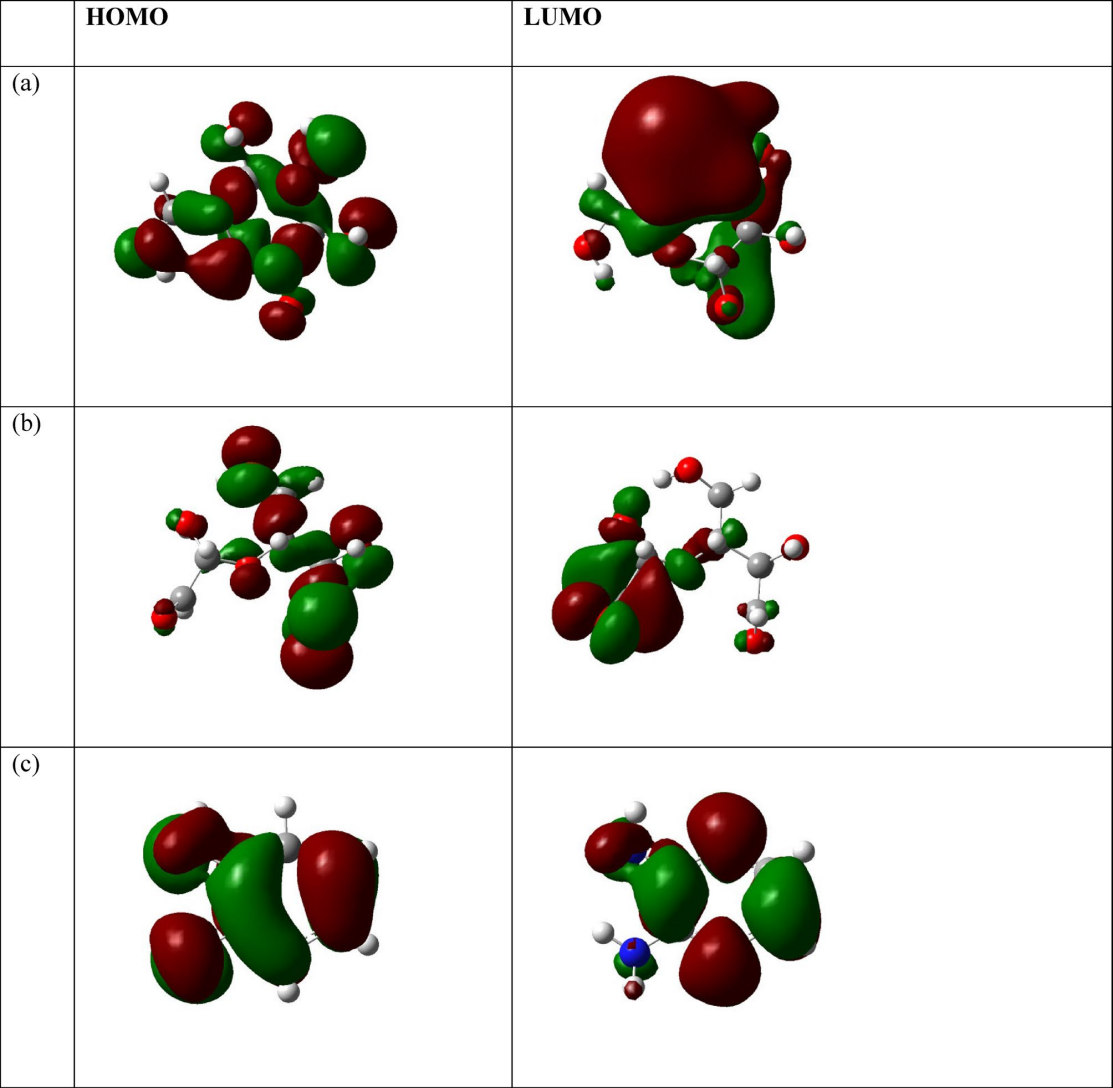

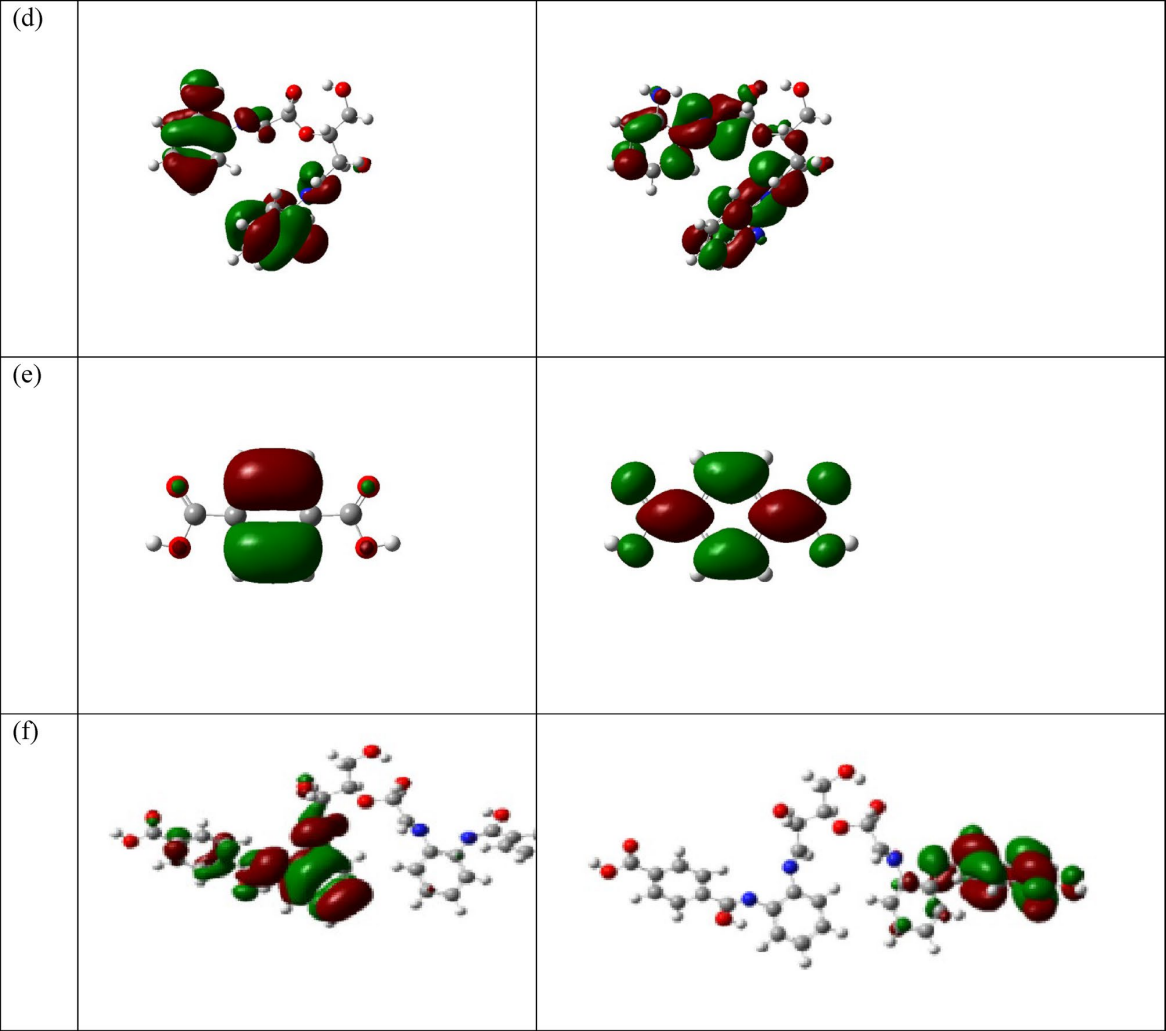
HOMO and LUMO structures for for (**a**) cellulose, (**b**) cellulose unit, PdA, (**c**) DAC, (**d**) DAC@PdA, (**e**) TPA and (**f**) DAC@PdA@TPA based on the DFT/B3LYP/6–31 g(d, p) methodology.

The Global Reactivity Parameters^91–93^ of a compound can be predicted from the HOMO-LUMO gap. The HOMO is electron donor and LUMO the electron acceptor sites are shown in Fig. (12) for the cellulose unit, DAC, PdA, DAC@PdA, TPA, and. From Table (4), the higher reactivity of the modified cellulose with terephethalic acid (DAC@PdA@TPA) over the cellulose is explained in the light of energy gap, ΔE_gap_, which measures the reactivity; as the energy gap decreases the reactivity increases. Table (4) illustrates that, ΔE_gap_ for the modified cellulose (DAC@PdA) was found to be more reactive than of the cellulose and the modified cellulose with terephethalic acid (DAC@PdA@TPA)also was found to be more reactive than the (DAC@PdA). (Hard molecules $\:\eta\:)$ have a large energy gap, and soft molecules$\:\sigma\:$)^94,95^. A soft molecule is more reactive than a hard molecule because a soft molecule has a lower ΔE _(LUMO−HOMO)_^71^. As seen from Table (4) the modified cellulose (DAC@PdA) is softer than of the cellulose and the modified cellulose with terephethalic acid (DAC@PdA@TPA)also is softer than the (DAC@PdA). This confirm that the modified cellulose (DAC@PdA) is more reactive than the cellulose and the modified cellulose with terephethalic acid (DAC@PdA@TPA)also is more reactive than the (DAC@PdA). $\:\chi\:$ is a measure of power of atom(s) to attract the electrons from the other molecules]^96^. A high value of electronegativity (χ) for the (DAC@PdA) suggests strong ability to attract electrons from the modified cellulose with terephethalic acid (DAC@PdA@TPA), which leads to greater interaction to form the compound.

The ionization potential; I_P_ and the electron affinity; E_A_, can be expressed as negative values of E_HOMO_ and E_LUMO_, respectively. Ionization energy is a descriptor expressing the chemical reactivity of atoms and molecules. Higher values of ionization energy indicate higher stability and chemical inertness and vice versa smaller ionization energy indicates higher reactivity of the atoms and molecules^97^. Table 4 shows the values of the ionization energy of the investigated molecules. The low ionization energy of the modified cellulose (DAC@PdA) indicates its high reactivity than cellulose and the modified cellulose with terephethalic acid (DAC@PdA@TPA)is high reactivity than the modified cellulose (DAC@PdA). According to the definition electrophilicity index (w) it measures the tendency of chemical species to acquire electrons. The results of electrophilicity shown in Table 5 are in decreasing order; **DAC@PdA@TPA** > **DAC@PdA** > **DAC** > **cellulose**. Finally, the dipole moment (µ) is a factor that can also provide information about interaction between molecules. The value (µ) of **DAC@PdA@TPA** is higher than (µ) of the **DAC@PdA** or cellulose unit, this suggests the stronger interactions between the **DAC@PdA** and **TPA** to form the **DAC@PdA@TPA** compound. The (E_HOMO_), (E_LUMO_), energy gap DE_(LUMO−HOMO)_, ionization potential (IP), electron affinity (EA), hardness ($\:\eta\:)$, softness ($\:\sigma\:$), electronegativity ($\:\chi\:$), chemical potential $\:\left(\mu\:\right)$, The Global electrophilicity index (w) and dipole moment of the cellulose unit, DAC, PdA, DAC@PdA, TPA and DAC@PdA@TPA are listed in Table 5.

**Table 4:** The global reactivity descriptors (GRD) determined using DFT/B3LYP/6–31 g(d, p) method of calculations for cellulose unit, DAC, PdA, DAC@PdA, TPA and DAC@PdA@TPA.

GRD)	Cellulose	DAC	phenylene diamine	DAC + phenylene diamine	t-phthalic acid	DAC + phenylene diamine + t-phthalic
E_HOMO_ [eV]	−0.25874	−0.25584	−0.18226	−0.19627	−0.27204	−0.21713
E_LUMO_ [eV]	−0.04166	−0.05246	0.018	−0.04275	−0.07784	−0.07482
ΔE_gap_ [eV]	0.21708	0.20338	0.20026	0.15352	0.1942	0.14231
η [eV]	0.10854	0.10169	0.10013	0.07676	0.0971	0.071155
σ [eV]^−1^	9.213193	9.833809	9.987017	13.02762	10.29866	14.05383
I_p_ [eV]	0.25874	0.25584	0.18226	0.19627	0.27204	0.21713
E_A_ [eV]	0.04166	0.05246	−0.018	0.04275	0.07784	0.07482
χ [eV]	0.1502	0.15415	0.08213	0.11951	0.17494	0.145975
µ [eV]	−0.1502	−0.15415	−0.08213	−0.11951	−0.17494	−0.145975
w [eV]	0.103925	0.1168366	0.0336829	0.0930344	0.1575901	0.1497344
The dipole moment [Debye]	1.8721	1.4728	2.3873	2.5009	2.6964	5.6771

#### Theoretical infrared analyses

**Fig. 13:**
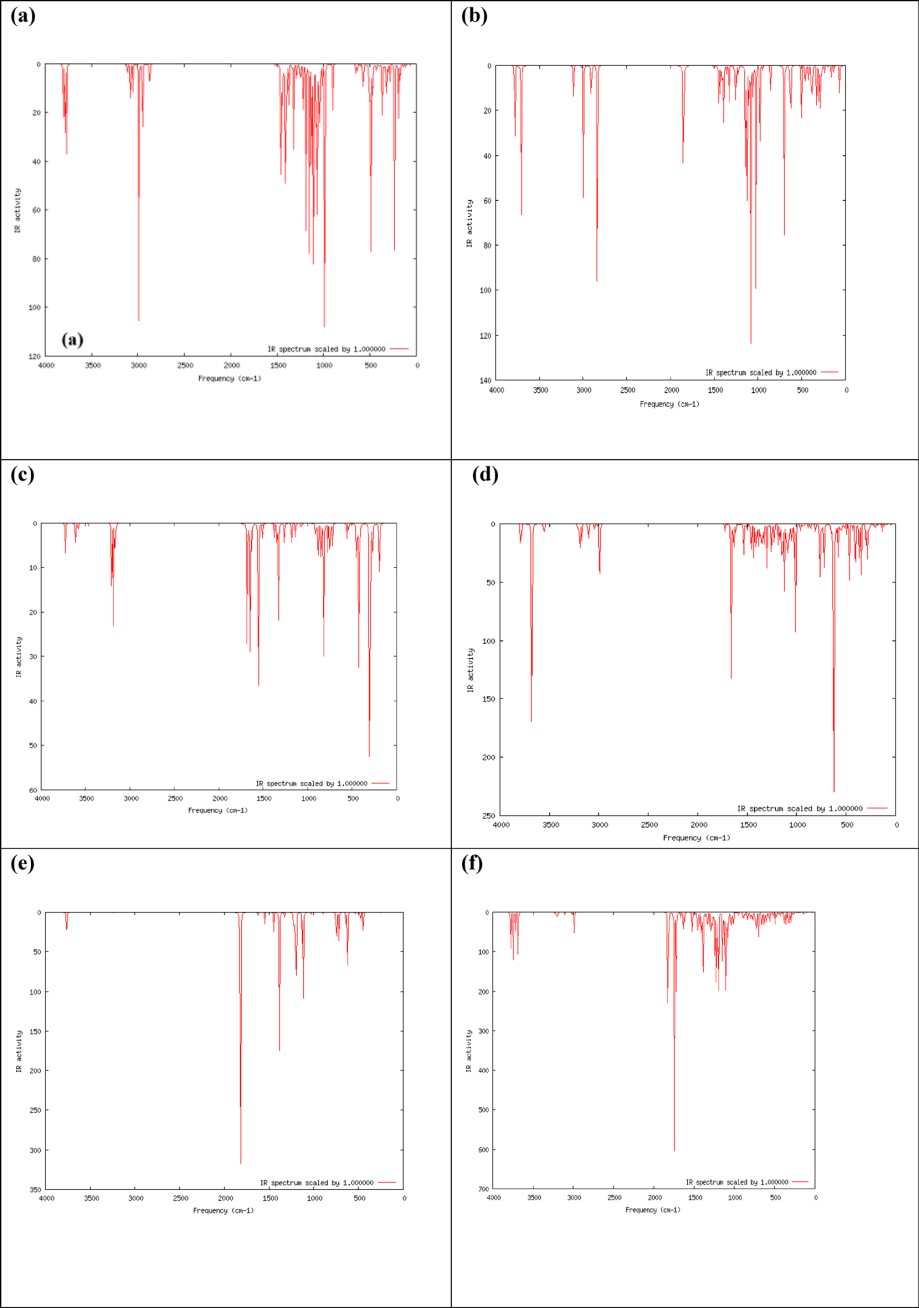
The calculated IR spectrum for (a) cellulose unit, (b) DAC, (c) PdA, (d) DAC@PdA, (e) TPA and (f) DAC@PdA@TPA based on the DFT/B3LYP/6–31 g(d, p) methodology.

Infrared analyses are supported by theoretical calculations that allow a trustworthy interpretation of experimental spectra of gaseous molecules. B3LYP/6–31 g(d, p) calculations are the most prominent method used to model IR spectra. The comparison is made between IR spectra calculated for gas phase molecules and those measured in the solid state. Furthermore, Asadi et al. report a comparison between gas phase DFT and condensed phase experimental data and obtain quite good agreement^98^. The IR spectrum of the cellulose unit, DAC, PdA, DAC@PdA, TPA and DAC@PdA@TPA are shown in Fig. (13).

#### Electrostatic potential (ESP)

**Fig. 14:**
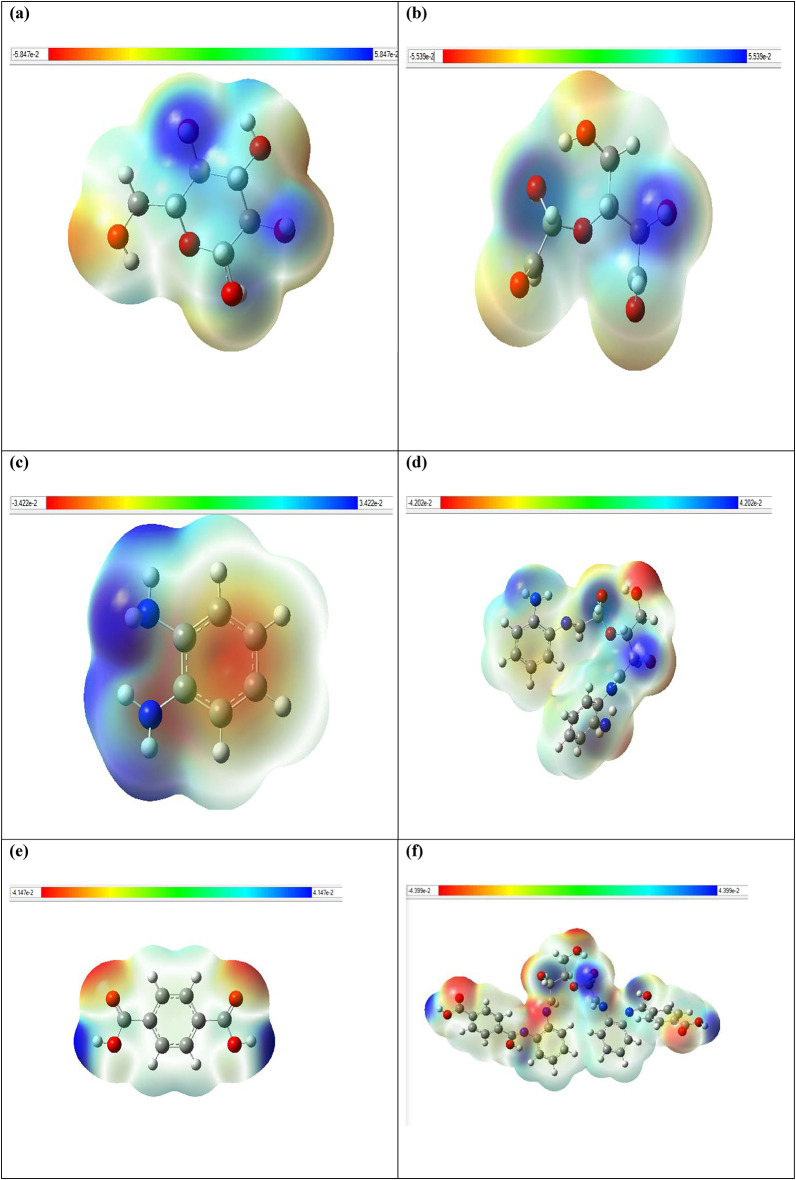
Electrostatic potential (ESP) surface maps visualization for (a) cellulose unit, (b) DAC, (c) PdA, (d) DAC@PdA, (e) TPA and (f) DAC@PdA@TPA based on the DFT/B3LYP/6–31 g(d, p) methodology. Blue, green and red correspond to ESP varying from min to max level, the blue and red spheres correspond to ESP surface minima and maxima, respectively. ESP ranges are included in the legend at each Figure panel.

An electrostatic potential (ESP) surface or map of a molecule shows the partial distribution of change along the molecule’s surface. Electrostatic potential maps, also known as electrostatic potential energy maps, or molecular electrical potential surfaces, illustrate the charge distributions of molecules three dimensionally. These maps allow us to visualize variably charged regions of a molecule. Knowledge of the charge distributions are very useful to help determine molecular polarity and can be used to determine how molecules interact with one another. To make the electrostatic potential energy data easy to interpret, a color spectrum, with red as the lowest electrostatic potential energy value and blue as the highest, is employed to convey the varying intensities of the electrostatic potential energy values. The calculated ESP maps of the cellulose unit, DAC, PdA, DAC@PdA, TPA and DAC@PdA@TPA are shown in Fig. (14).

### Biological activity studies

The study tested the antimicrobial activity of the DAC@PdA@FM (MOF) against three Gram-negative bacterial strains (*E. coli*, *Enterococcus colaceae*, and *Klebsiella pneumoniae*) and two Gram-positive strains (*Bacillus subtilis* and *Staphylococcus aureus*) using the agar diffusion method as shown in Fig.S2. The effectiveness of the MOF:DAC@PdA@FM nanocomposite was measured by the inhibition zone diameter, against positive control (Azithromycin) antibiotic. As shown in Table 5, the inhibition zone of *E. coli*, *Enterococcus colacae* and *Klebsiella pneumoniae* are 19 mm, 13 mm and 14 mm, respectively. No inhibition observed for *S. aureus* and *B. subtilis*. The high antibacterial activity of the examined MOF:DAC@PdA@FM nanocomposite is linked to its properties, including high surface area, structure, and porosity^99^. The MOF:DAC@PdA@FM nanocomposite showed antibacterial activity only against Gram-negative bacteria, with no inhibition effect observed on Gram-positive strains. This selective behavior may result from the structural differences between the two types. Gram-negative bacteria have an outer membrane that enhances interaction with the composite and allows easier penetration and possible generation of reactive oxygen species (ROS) but the effect was significantly weaker than the control antibiotic. Similar selectivity has been reported in other MOF-based materials used for antimicrobial purposes^100–102^.

**Table 5:** Antimicrobial activity of the DAC@PdA@FM (Data obtained as the diameter of zone of Inhibition, mm).

Samples	E.coli	Entero	Klebsiella	S. aureus	B. sub
**MOF:DAC@PdA@FM nanocomposite **	19 mm	13 mm	14 mm	-ve	-ve
**Control** **(DMSO)**	18 mm	-ve	13 mm	-ve	-ve
**Antibiotic** **(Azithromycin)**	29 mm	-ve	-ve	24 mm	24 mm

### Photocatalytic studies

#### **Point of zero charge pH**_**PZC**_

FigureS3 demonstrates the point of zero charge of the MOF:DAC@PdA@FM nanocomposite to realize the mechanism of dyes removal by the composite. It is indicated that at pH 5.33 the surface charge of the nanocomposite is neutral. Below this pH, the material’s surface is positively charged, and above this pH, it becomes negatively charged. Cationic dyes (CV and TBO ) will be adsorbed more effectively at higher pH values (above **pH**_**PZC**_), where the surface becomes negatively charged. But anionic Dye (E110) is adsorbed at lower pH values (below **pH**_**PZC**_), where the surface is positively charged.

#### Influence of pH

**Fig. 15:**
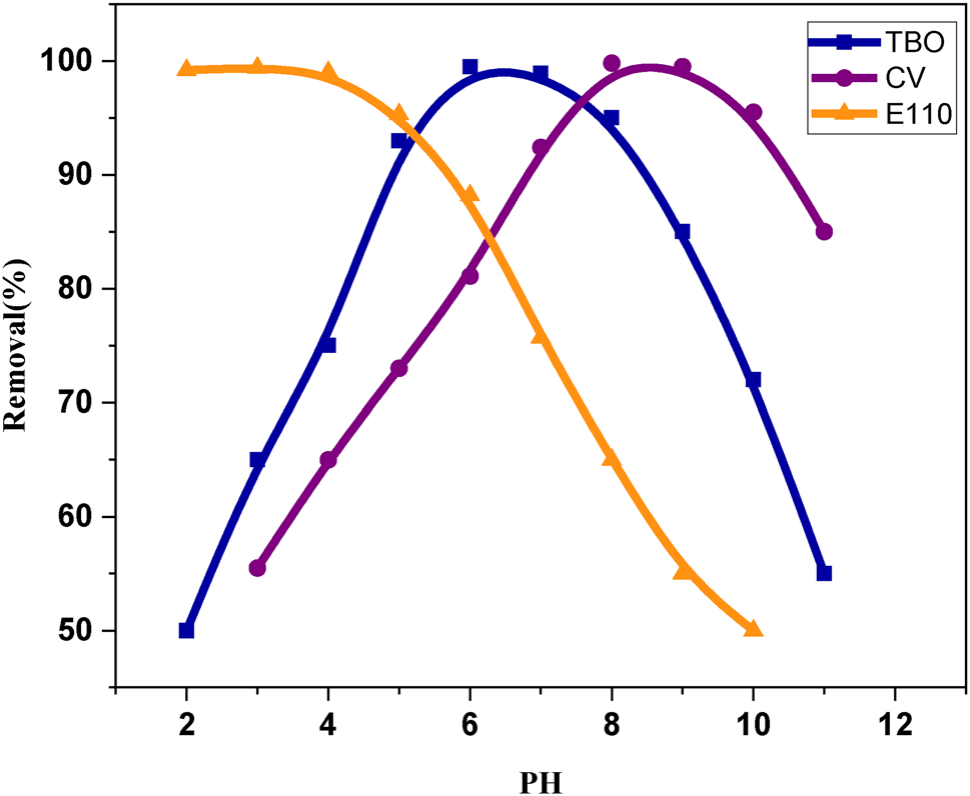
Effect of pH on the degradation of (TBO, CV and E110) cationic and anionic dyes by the MOF:DAC@PdA@FM nanocomposite.

The study investigates how pH affects the degradation of dyes using the MOF:DAC@PdA@FM nanocomposite. It highlights that pH influences the surface charge of the nanocomposite and the degree of dye dissociation. The effect of pH on the degradation of dyes using the MOF:DAC@PdA@FM nanocomposite was investigated within a pH range of 2–8. The results in Fig. 15 indicated that the sorption-photocatalytic degradation efficiency, increased with a pH up to 6 and 8 TBO and CV, respectively, after which it declined. Conversely, for E110, degradation improved as the pH decreased to 3.The effective removal of cationic dyes, at higher pH, is attributed to strong electrostatic interactions between the MOF:DAC@PdA@FM and dye molecules. At acidic pH, reduced dye removal occurs due to excess H^+^ ions competing with cationic dyes for sorption sites, though E110 shows higher adsorption due to electrostatic attraction^103–105^. The adsorption behavior was strongly pH-dependent. At higher pH, the surface became negatively charged, enhancing uptake of cationic dyes. At lower pH, competition with H⁺ reduced adsorption of cationic dyes but improved binding with anionic E110. These changes influenced both equilibrium capacity and adsorption rate.

#### Influence of the photocatalyst dosage

The analysis of catalyst dose indicated that the percentage degradation of the three dyes improved as the weight of the MOF:DAC@PdA@FM nanocomposite increased from 0.0025 to 0.005 g^106^. However, the amount of adsorbed dyes decreased with higher adsorbent dosage beyond this range as in Fig.S4.

#### Influence of initial concentration of dyes and sorption–photodegradation isotherms

The study investigated the effect of initial dye concentration on the sorption and photodegradation of dyes using DAC@PdA@FM. From Fig.S5, the results indicate optimal removal concentrations for E110, TBO, and CV are 100, 200, and 250 mg/L, respectively. The equilibrium sorption capacity of DAC@PdA@FM increases with higher initial dye concentrations due to a greater driving force at the solid-liquid interface. Initially, adsorption is rapid but slows as equilibrium is approached, eventually plateauing as active sites become saturated. While high adsorption improves photo degradation, excess dye concentration can saturate the nanocomposite and hinder light penetration, reducing photo degradation efficiency.

**Fig. 16:**
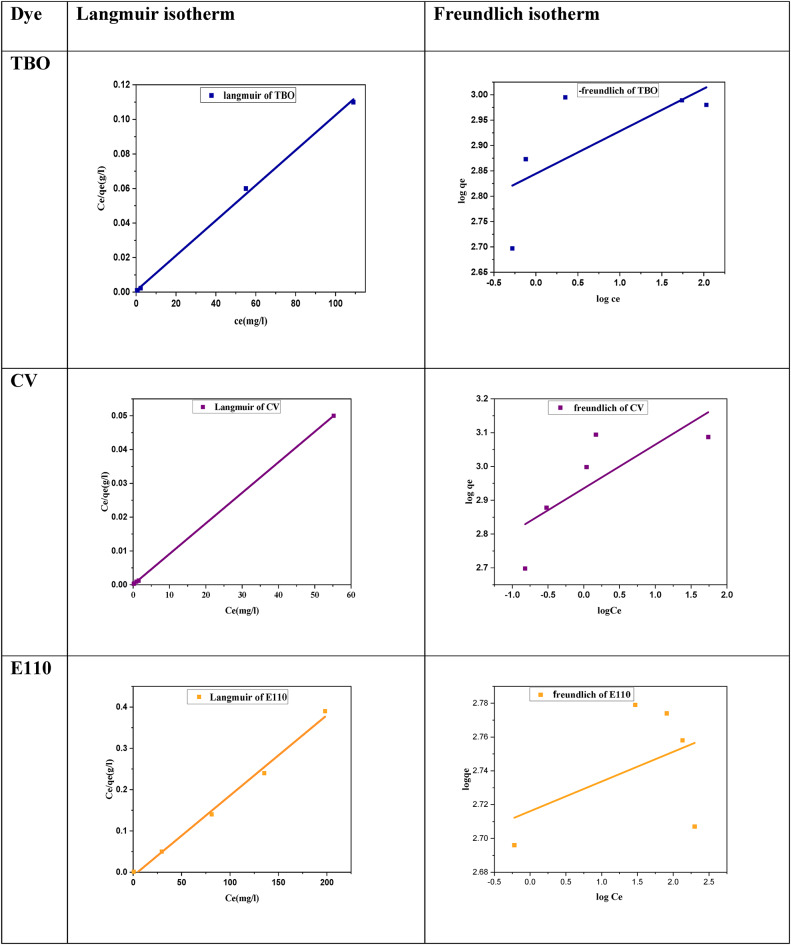
Photodegredation isotherms for TBO, CV and E110 degradation by DAC@PdA@FM: (a) Langmuir isotherm model, (b) Freundlich isotherm model.

The adsorption isotherms of the MOF: DAC@PdA@FM nanocomposite for three dyes were analyzed using the Langmuir and Freundlich models to correlate equilibrium data in sorption system design (Fig. 16). The models indicate how adsorbed molecules distribute between liquid and solid phases at equilibrium, with better regression coefficients (*R²*) showing stronger model applicability. The Langmuir model assumes monolayer adsorption on homogeneous surface sites, while the Freundlich model accounts for non-ideal sorption on heterogeneous surfaces^107^. Linear forms of both isotherm equations are presented in Eq. 11.

##### **Separation factor**

A crucial characteristic of Langmuir isotherm can be estimated by a dimensionless constant called equilibrium parameter, R_L_^108^, defined by:

11 $$\:{\mathbf{R}}_{\mathbf{L}=\frac{1}{1+{\mathbf{K}}_{\mathbf{L}}.{\varvec{C}}_{\varvec{i}}}}\:$$

Where C_i_ is the maximum initial dye concentration (mg/L), and R_L_ values indicate the type of isotherm to be either, favorable (0 < R_L_), linear (RL = 1), or unfavorable (R_L_>1). As seen in fig, the adsorption of dyes TBO, CV, and E110 on DAC@PdA@FM was analysed using Langmuir and Freundlich models, with the Langmuir model showing a better fit (higher R² values) than Freundlich which was confirmed through the experimental data obtained in Table 6.

**Table 6:** Langmuir and Freundlich isotherm parameters and error analysis for (TBO, CV and) dyes degradation by MOF:DAC@PdA@FM nanocomposite.

Langmuir isotherm										
**Dye**	**q**_**max**_ **(mg/g)**		**K**_**L**_	**R**_**L**_	**R** ^**2**^	$\:{\varvec{x}}^{2}$	**SSE**	**MSE**	**Hybrid**	$\:\varDelta\:\varvec{q}\varvec{\%}$
**TBO**	980.392		1.294	3.85 × 10^−3^	0.99928	294.63	288853.66	57770.73	18108.24	26.05
**CV**	1105.43		15.07	2.654 × 10^−4^	0.99996	488.313	539795.84	107959.16	31499.21	43.38
**Freundlich isotherm**										
**Dye**	**N**	**K**_**F**_			**R** ^**2**^	$\:{\varvec{x}}^{2}$	**SSE**	**MSE**	**Hybrid**	$\:\varDelta\:\varvec{q}\varvec{\%}$
**TBO**	11.93	698.92			0.32868	372.442	385676.47	77135.294	23572.48	33.35
**CV**	7.73	861.41			0.46055	1157.21	1672371.7	334474.34	90562.26	117.98

In addition, the R_L_ values were consistently less than 1, indicating a favourable adsorption process. The maximum degradation efficiencies were 988.75 mg/g, 98.9% for TBO, 1242.5 mg/g,99.4% for CV, and 497 mg/g 99.4% for E110. Overall, the results imply that the adsorption process follows the Langmuir isotherm model, suggesting specific monolayer binding of each dye to the DAC@PdA@FM surface^109^.The accuracy and predictive performance of the adsorption and kinetic models were further evaluated using statistical error functions, including SSE, MSE, and the Hybrid error function, as defined in Sect. 2.5.6. These parameters enabled comparison between experimental and theoretical values to determine the best-fitting models. The comparison between Langmuir and Freundlich models, along with error function analysis (χ^2^, SSE, MSE, Hybrid), showed agreement across all dyes. All three dyes demonstrated high R² values with the Langmuir model (TBO: 0.99928, CV: 0.99996, E110: 0.99238), indicating that adsorption occurred mainly through monolayer formation on active sites. In contrast, the Freundlich model gave significantly lower or even negative R² values, confirming it was less suitable for describing the adsorption process in this system. Moreover, the error functions (χ^2 ^, SSE, MSE, Hybrid) were consistently lower in the Langmuir model compared to Freundlich, despite small variations among the dyes. This confirms that the statistical fitting supports the experimental trend, where adsorption occurred at well-defined active sites on the surface of the DAC@PdA@FM composite.

#### Reaction time and photocatalytic kinetic studies

**Fig. 17:**
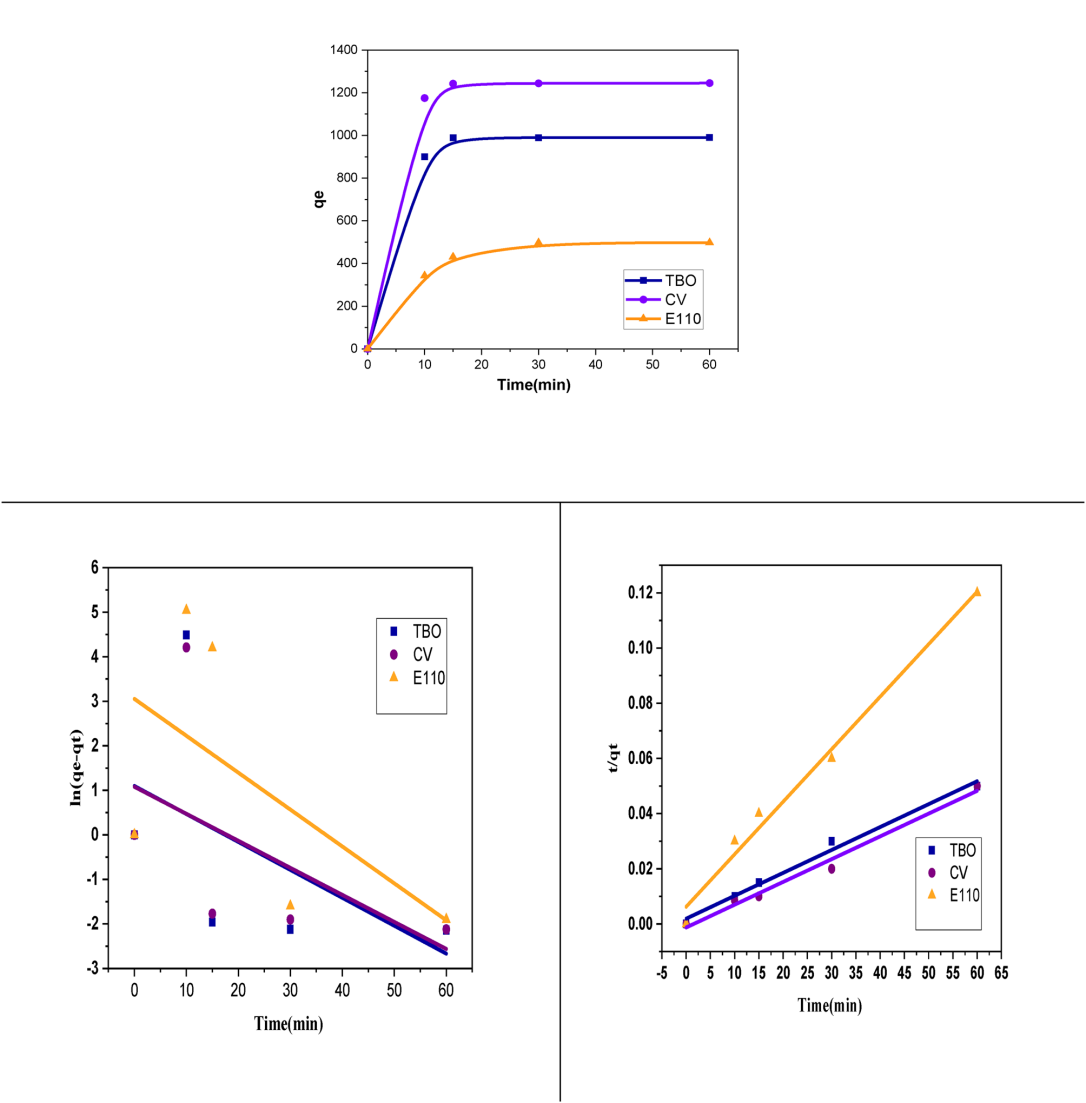
(a) Effect of time on TBO, CV andE110 sorption on MOF: **DAC@PdA@FM nanocomposite **, (b) Pseudo-1 st-order for TBO, CV and E110 sorption (c) Pseudo-2nd-order for TBO, CV and E110 sorption.

Contact time significantly influences the adsorption kinetics of DAC@PdA@FM for three dyes (TBO, CV, and E110). As shown in Fig. 17, the adsorption capacity of the MOF:DAC@PdA@FM nanocomposite increased rapidly from 10 to 60 min, with over 90% of equilibrium reached within 30 min. After 1 h, the capacity stabilized, indicating equilibrium. Among the dyes, CV demonstrated the highest adsorption rate, while E110 showed the lowest. The adsorption reached equilibrium within 30 min for most dyes. Detailed kinetic modeling is discussed in Table 7. This process prominently features the cationic dyes (CV and TBO), where initial adsorption is influenced primarily by electrostatic attraction. Equilibrium time for dye adsorption varies with the composite and initial dye concentration, typically reaching equilibrium within 30 min. However the Anionic dye E110, show rapid adsorption in acidic conditions due to their strong attraction to positively charged surfaces. The equilibrium time for anionic dyes may also depend on pH and composite properties but is generally comparable to cationic dyes^110^.

**Table 7:** Kinetic parameters and error analysis for pseudo-first-order and pseudo-second-order models for (TBO, CV and E110) dyes degradation by DAC@PdA@FM.

Pseudo first order kinetic model										
**Dye**	**q**_**e**_ **(mg/g)**	**K**_**1**_ **(min** ^**−1**^ **)**	**R** ^**2**^	$\:{\varvec{x}}^{2}$	**SSE**		**MSE**	**Hybrid**		$\:\varDelta\:\varvec{q}\varvec{\%}$
**TBO**	2.99	−0.00104	0.01864	1245464.164	3723937.85		930984.4625	192223.352		132.5087566
**CV**	2.95	−0.001011	0.04285	2031552.105	5993078.71		1498269.678	244130.4586		132.6923897
**E110**	21.19	−0.001380	0.13481	34169.002	724041.1644		181010.2911	80107.00022		120.5748011
**Pseudo second order kinetic model**										
**Dye**	**q**_**e**_ **(mg/g)**	**K**_**2(min**_^**−1**^_**)**_	**R** ^**2**^	$\:{\varvec{x}}^{2}$	**SSE**	**MSE**		**Hybrid**	$\:\varDelta\:\varvec{q}\varvec{\%}$	
**TBO**	1204.384	3.63 × 10^−4^	0.9891	191.998	231240.4075	57810.10188		12150.6	8.531506025	
**CV**	1211.39	−5.28 × 10^−4^	0.98195	3.702447932	4485.1084	1121.2771		183.4	0.100067162	
**E110**	526.32	5.80 × 10^−4^	0.98337	84.85620839	44661.5196	11165.3799		6169.151284	11.48083161	

Quantitative kinetic analyses are essential for designing efficient degradation systems and understanding the degradation mechanisms, especially for identifying the rate-limiting steps of photocatalytic degradas of dye degradation and adsorption studied by DAC@PdA@FM are shown in (Fig. 16). The parameters of pseudo-first-order and pseudo-second-order kinetic models given for TBO, CV and E110 adsorption by the MOF:DAC@PdA@FM are listed in Table 7. For three dyes analyzed, the regression coefficient (R²) values ranged from 0.01264 to 0.13481 for the pseudo-first-order model and 0.98337–0.9891 for the pseudo-second-order model. The higher R² values for the pseudo-second-order model indicate a better fit with experimental data. The experimental maximum capacities for the dyes are closer to the values predicted by the second-order model (q_e_2) than the first (q_e_1). The low R² values and high rate constants from the first-order model indicated a poor fit and slow adsorption kinetics^88^. The dye degradation data fit better with the pseudo-second-order model (R² > 0.99), suggesting chemisorption as the rate-limiting step involving electron transfer or sharing between dye molecules and active sites on the composite surface. For all dyes in error analysis, the pseudo-second-order kinetic model gave the best fit, with the higher R² values (TBO: 0.9891, CV: 0.98195, E110: 0.98337) and the lower error functions values. This implies that the adsorption mechanism is governed primarily by chemisorption, involving electron exchange or specific interaction between the dyes and surface functional groups. These kinetic results strongly support the isotherm findings, as both models suggest specific, strong, and likely chemical adsorption rather than random, multilayer physical adsorption. These findings confirm that the statistical modeling outcomes are in strong agreement with the experimental observations, supporting the reliability and consistency of both the theoretical and practical aspects of the adsorption process.

#### Influence of temperature on the dyes degradation (thermodynamic studies)

The study investigates the thermodynamic parameters free energy (ΔG^o^), enthalpy (ΔH^o^), and entropy (ΔS^o^) related to the degradation of TBO, CV, and E110 dyes by the MOF:DAC@PdA@FM nanocomposite under light exposure, within a temperature range of 298 K to 318 K. The thermodynamic equilibrium constant (Kc) in addition to the rest of the thermodynamic parameters was calculated as in (Eq. 9) and (Eq. 10). The values obtained and all the thermodynamic parameters are estimated in Table 8 and the plotted Figures are shown in Fig.S6. As observed, the degradation process for CV and TBO is exothermic, as indicated by negative values of ΔΗ^o^, suggesting heat loss to the solution. In contrast, the positive ΔH° value observed for E110 submits an endothermic sorption process.The ΔH^o^ values for CV and TBO range from − 40 to −400 Kjoule/mole, indicating that their adsorption involves chemisorption, while the smaller value for E110 aligns more with physisorption. In addition, Positive entropy (ΔS^o^) implies increased disorder, as seen with E110 dye, while negative entropy proposes decreased disorder for CV and TBO dyes. The negative Gibbs free energy (ΔG°) confirms the spontaneous and thermodynamically favorable nature of the dye sorption process. The endothermic nature of E110 degradation proves that the reaction requires energy input to proceed. This is likely due to the structural stability of E110 and the presence of sulfonate groups, which make it less reactive compared to CV and TBO. These cationic dyes, on the other hand, undergo spontaneous degradation with energy release, as reflected in their exothermic profiles. The differences in charge, molecular structure, and surface interaction behavior explain the contrasting thermodynamic responses observed.Furthermore, it notes that the degradation of CV and TBO is more favorable at lower temperatures, as higher temperatures lead to a decrease in degradation efficiency^111,112^.

**Table 8:** Thermodynamic parameters for the sorption of TBO, CV and E110 onto the MOF:DAC@PdA@FM nanocomposite.

Dye	T (k)	K_c_	ΔG^o^ (KJ/mol)	ΔH^o^ (KJ/mol)	ΔS^o^ (J/mol K)	*R* ^2^
**TBO**	**298**	365.037	−14.618	−189.627	−588.07	0.99999
	**308**	29.964	−8.706			
	**318**	3.03435	−2.935			
**CV**	**298**	73.699	−10.654	−140.032	−434.508	0.99811
	**308**	12.6796	−6.504			
	**318**	2.1382	−1.946			
**E110**	**298**	837.147	−16.674	3.956057	69.23932	0.99849
	**308**	880.0687	−17.362			
	**318**	925.1908	−18.058			

#### Effect of ionic strength

The study examined the influence of the interaction of Na^+^ and Cl^−^ ions using NaCl as the inorganic electrolyte. Specifically, 0.005 g of MOF:DAC@PdA@FM nanocomposite was added to 25 ml of aqueous solutions containing E110, TBO, and CV at concentrations of 100, 200, and 250 ppm, respectively. The experiments were conducted at 25 °C for 30 min to assess the effects of different electrolyte concentrations ranged between 10 and 100 mM. Results in Fig.S7 showed that the adsorption efficiency declined for cationic dyes, while it remained constant for anionic dyes as electrolyte concentration increased. It was achieving removal efficiencies of about 91.7%, 92.3%, and 90.1% for TBO, CV, and E110, respectively. This behavior can be explained by electrostatic competition. As NaCl concentration increases, Na⁺ ions compete with the cationic dyes (CV and TBO) for the negatively charged adsorption sites on the composite surface. This reduces dye uptake. Unlike, the anionic dye E110 is adsorbed under acidic conditions where the surface becomes positively charged. In such a case, Cl⁻ ions from NaCl do not interfere with adsorption, so E110 removal remains unaffected.

### Desorption and reusability studies

The recyclability of photocatalyst is a significant challenge during degradation processes. The DAC@PdA@FM can be easily separated from solutions using an external magnetic field, making it recyclable and reusable. The reusability studies were conducted using various eluents (Ethanol, 0.1 NaOH, and 0.1 Na_2_CO_3_ to extract the dyes from MOF: DAC@PdA@FM nanocomposite. Among the tested options, 0.1 M NaOH was the most effective for desorption. The MOF:DAC@PdA@FM demonstrated high reusability, maintaining sorption efficiency above 89% across four cycles of sorption and desorption as in Table 9. That is indicating its potential as a sorbent for both cationic and anionic dye removal from aqueous solutions even without washing between cycles^24^. The superparamagnetic nature of the MOF:DAC@PdA@FM nanocomposite provides significant practical advantages in water treatment. It enables rapid and efficient magnetic separation of the composite from treated water without requiring filtration or centrifugation. Since the material exhibits no residual magnetism, it avoids aggregation and remains easily redispersible for repeated use. These properties enhance operational simplicity, reduce processing time, and support integration into continuous flow systems for real-world wastewater applications.

**Table 9:** Repeated degradation of TBO, CV and E110 dyes using MOF: DAC@PdA@FM regeneration.

Dye	Cycle	Desorption (%)	Recovery (%)
TBO	1	97.8	96.3
	2	96.5	96.0
	3	93.1	91.9
	4	90.9	89.3
CV	1	98.9	97.5
	2	96.8	95.2
	3	95.6	93.9
	4	94.1	92.4
E110	1	95.1	93.8
	2	94.2	92.7
	3	93.3	91.5
	4	92.0	89.1

### Application

The MOF:DAC@PdA@FM nanocomposite was tested for its ability to remove dyes from various water samples, including tap water, seawater, and wastewater, at concentration of 150 mg/L. In real wastewater samples, the removal efficiency for CV and TBO remained above 96%, while for E110 it decreased to around 75%. This reduction may result from matrix interference and competition with other contaminants in real water. Compared to CV and TBO, the removal efficiency of E110 in real water samples was significantly lower. This reduction is likely due to the presence of competing anionic species and natural organic matter in wastewater, which interferes with E110 binding on the composite surface. Since E110 is an anionic dye, it faces electrostatic repulsion from negatively charged components in the matrix and reduced accessibility to adsorption sites. These effects were not observed with cationic dyes, which interacted more favorably with the composite under the same conditions.The result is indicating the composite’s effectiveness for organic dye removal in practical applications, as shown in Table 10.

**Table 10:** Analytical results of cationic and anionic dyes photocatalytic degradation (µg ml^−1^) in real water samples using the MOF: DAC@PdA@FM nanocomposite.

Type of dye	Water samples type & location	Added (µg/mL)	Found (µg/mL)	Recovered (µg/mL)	Recovery (%)
**CV**	**Tap water** **(Mansoura university, Mansoura, Egypt)**	0.00	0.00	0.00	0.00
		**150**	**2.7**	**147.3**	**98.2**
	**Wastewater(Sinbellawin sewage station, Dakahlia**	0.00	0.00	0.00	0.00
		**150**	**3.1**	**146.9**	**97.9**
	**Sea water** **(Marsa Matrouh, Egypt)**	0.00	0.00	0.00	0.00
		**150**	**3.2**	**146.8**	**97.8**
**TBO**	**Tap water** **(Mansoura university**,** Mansoura**,** Egypt)**	0.00	0.00	0.00	0.00
		**150**	**4.8**	**145.2**	**96.8**
	**Wastewater** **(Sinbellawin sewage station**,** Dakahlia**,	0.00	0.00	0.00	0.00
		**150**	5.2	144.8	96.5
	**Sea water** **(Marsa Matrouh**,** Egypt)**	**0.00**	0.00	0.00	0.00
		**150**	5.4	144.6	96.4
**E110**	**Tap water** **(Mansoura university**,** Mansoura**,** Egypt)**	**0.00**	**0.00**	**0.00**	**0.00**
		**150**	**36.2**	**113.8**	**75.8**
	**Wastewater** **(Sinbellawin sewage station**,** Dakahlia**,	0.00	0.00	0.00	0.00
		**150**	37.08	112.92	75.3
	**Sea water** **(Marsa Matrouh**,** Egypt)**	**0.00**	0.00	0.00	0.00
		**150**	37.3	112.7	75.1

### Performance of the novel MOF: DAC@PdA@FM nanocomposite 

As presented in Table 11, the MOF:DAC@PdA@FM nanocomposite outperformed several previously reported MOF-based photocatalysts in terms of removal efficiency, required catalyst dose, and reaction time. While other systems often depend on larger doses and extended treatment durations, DAC@PdA@FM achieved high degradation efficiency (> 98%) for multiple dyes using only 5 mg of catalyst within 30 min. Its magnetic property allowed for fast and simple separation using an external magnet, eliminating the need for filtration or centrifugation, which are common in non-magnetic MOFs. Additionally, the composite retained its photocatalytic activity over four successive reuse cycles with minimal efficiency loss. These findings highlight the practical advantages of the MOF:DAC@PdA@FM nanocomposite in terms of operational ease, cost-effectiveness, and suitability for continuous water treatment applications.

**Table 11:** Comparison on photocatalytic activity of the MOF:DAC@PdA@FM nanocomposite with other MOF-based photocatalysts for dye degradation.

Photocatalyst	Dyes	Dye conc. (ppm)	Catalyst dosage (mg)	Time (min)	Degradation efficiency (%)	Reusability (cycles)	Magnetic property	Separation method	Ref.
**Fe**_**3**_**O**_**4**_ **@MOF-5**	MB	100	20	60	45	5	Yes	External magnet	^86^
**MIL-53 Fe@MIL-53 Sr**	RhB	20	100	60	67	–	No	filtration	^113^
**BUT-206**	CV	5	50	120	92.5	5	No	Filtration	^114^
**Co-(TIPE)(m-H2BDC)**	MO	6.5	20	250	95	3	No	Filtration	^115^
**Ag/Agcl@ZIf-8**	CV	25	30	90	90	6	No	Filtration	^116^
**Ti-MOF(MIL-125(Ti))**	RhB	10	50	150	85	3	No	Filtration	^117^
**MOF-5/rGO**	MB RhB MO	32	20	20	93 97 92	4	No	Filtration	^118^
**MOF: DAC@PdA@FM nanocomposite**	**TBO** **CV** **E110**	**200** **250** **100**	**5**	**30**	**98.88** **99.43** **99.42**	4	**Yes**	**External magnet**	**This Work**

### Plausible mechanism of photocatalytic degradation of TBO, CV, and E110 using the MOF: DAC@PdA@FM nanocomposite

The possible mechanism of TBO, CV, and E110 photocatalytic degradation on the surface of the MOF: DAC@PdA@FM nanocomposite has been clarified. MOFs have high surface area and porous nature that enable them to act as impressive light harvesters. When photons are absorbed by the MOF: DAC@PdA@FM nanocomposite after exposure to UV light source, electrons are excited from the valence band to the conduction band, leading to the formation of electron-hole pairs. The excited electrons reduce molecular oxygen to form superoxide radicals (•O₂⁻), while the holes oxidize water molecules, generating hydroxyl radicals (•OH), which are main agents in dye degradation.Cationic dyes are degraded at negatively charged sites on the MOF:DAC@PdA@FM nanocomposite by hydroxyl radicals, producing non-toxic by-products like CO₂ and H₂O. Anionic dyes bind to positively charged sites and are broken down by holes or superoxide radicals, resulting in the complete mineralization of dye molecules into harmless substances that help in water detoxification^119^. The photocatalytic mechanism contains five stages including (Eqs. (12)–(18)). In conclusion, the high photocatalytic efficiency of the MOF: DAC@PdA@FM nanocomposite is attributed to a combination of interaction mechanisms, including electrostatic attraction between the charged surface and dye ions, π–π stacking between aromatic dye rings and phenyl groups in PdA, hydrogen bonding with hydroxyl/carboxyl groups, and dye diffusion into porous structures (pore-filling effect). The electrostatic interactions between the charged surface and dye molecules support rapid adsorption, while π–π stacking between the aromatic structures of the dyes and PdA groups stabilizes binding and enhances charge transfer during photocatalysis.

12 $${\rm Photocatalyst (DAC@PdA@FM ) + light source (hv)\rightarrow\:e^{-}CB + h^{+}VB}$$

13 $${\rm e^{-}CB + h^{+}VB \rightarrow\:energy\: (heat)}$$

14 $${\rm O_{2} + e{-}CB \rightarrow\bullet\:O_{2}}$$

15 $${\rm H_{2}O + h^{+}VB\rightarrow\bullet\:OH + H^{+}}$$

16 $${\rm Cationic dye +\bullet\: OH \rightarrow CO_{2} + H_{2}O + other by- products}$$

17 $${\rm Anionic dye + h^{+} VB \rightarrow CO_{2} + H_{2}O + other by- products}$$

18 $${\rm Anionic dye +\bullet O_{2}^{-} \rightarrow CO_{2} + H_{2}O + other by- products}$$

## **Conclusion**

In this study, a novel magnetic cellulose-based MOF nanocomposite (DAC@PdA@FM) was successfully synthesized and applied for the removal of both cationic and anionic dyes from water. The composite showed strong adsorption and photocatalytic performance under visible light, with high removal efficiencies for crystal violet, toluidine blue O, and E110 dyes. Its performance was influenced by several factors, including pH, dye concentration, dosage, contact time, and temperature. The presence of Fe₃O₄ nanoparticles gave the composite magnetic properties, allowing for easy separation from solution using an external magnet. This feature enabled rapid recovery and reuse of the photocatalyst for at least five cycles, with minimal loss in activity. Kinetic studies showed that dye removal followed the pseudo-second-order model. While thermodynamic data confirmed that the adsorption processes were spontaneous and exothermic for the removal of TBO and CV and endothermic for E110 degradation. Furthermore, the adsorption and degradation data exhibited agreement with the Langmuir isotherm and pseudo-second-order kinetic models, as supported by the higher R² values and the lower error functions metrics, confirming the chemical nature and efficiency of the dye interaction mechanisms.

**Fig. 18:**
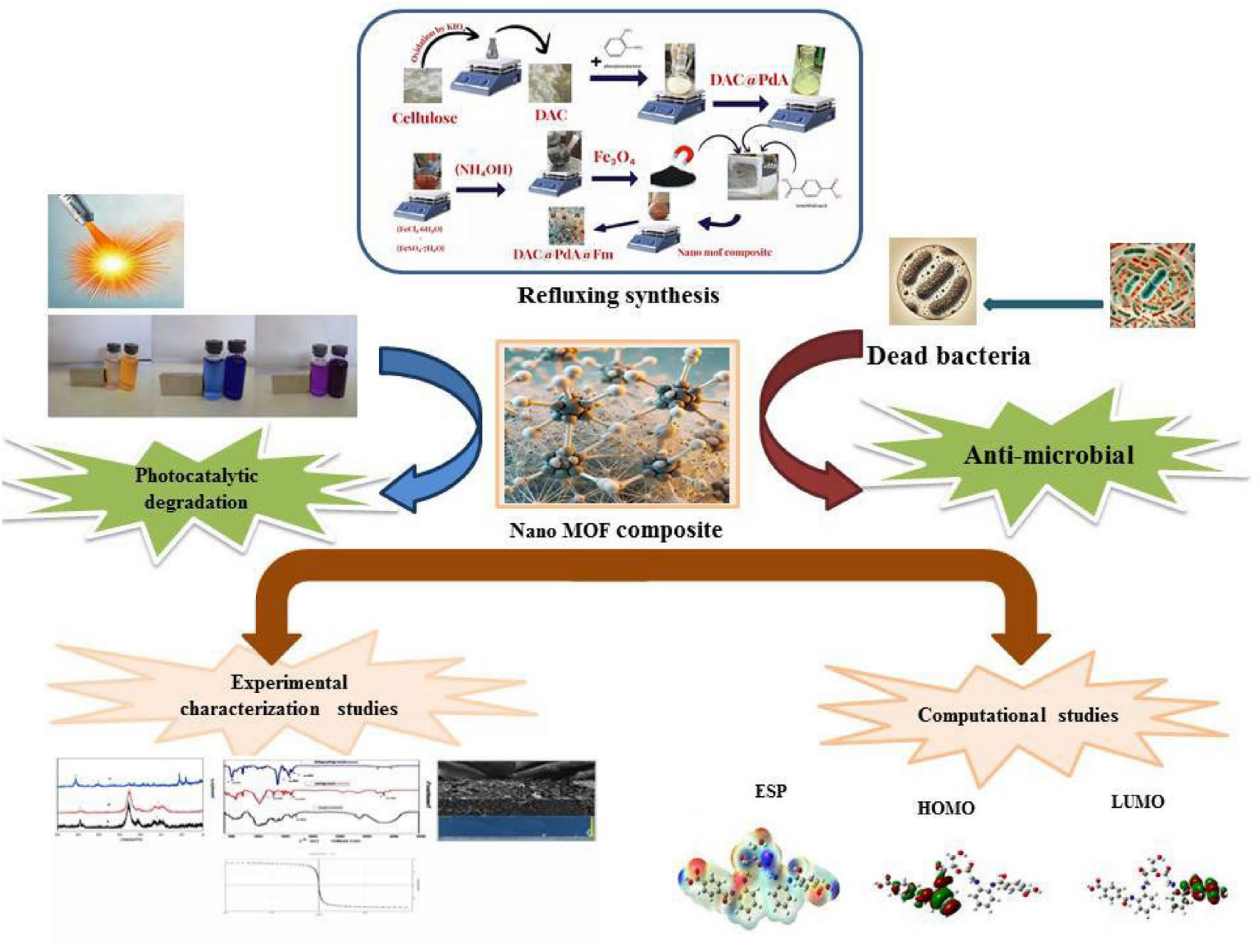
Schematic representation of synthesis, characterization and application of the MOF: DAC@PdA@FM nanocomposite.

Computational analyses supported the experimental findings and provided insights into the electronic structure and interaction mechanisms. The nanocomposite also showed weak but selective antibacterial activity, particularly against Gram-negative bacteria, suggesting potential for limited microbial control in water treatment. Finally, the MOF:DAC@PdA@FM nanocomposite combines high efficiency, reusability, and low-cost synthesis, making it a promising candidate for real-world applications in wastewater treatment. Further work is recommended to assess its performance in continuous flow systems and evaluate its long-term environmental safety. The synthesis, characterization and application of the MOF:DAC@PdA@FM nanocomposite is schematically represented in Fig. 18.

## Electronic supplementary material

Below is the link to the electronic supplementary material.

Supplementary Material 1

## Data Availability

Data is provided within the manuscript or supplementary information files.
