# Supplementary material for: Concurrent photocatalytic degradation of organic pollutants using smart magnetically cellulose-based metal organic framework nanocomposite
Source: Sci Rep. 2025 Jun 20;15:20100. doi: 10.1038/s41598-025-03256-5 (PMC12181425; doi:10.1038/s41598-025-03256-5)
Supplement: Supplementary file 1 — Supplementary Material 1 [file 41598_2025_3256_MOESM1_ESM.docx]

**Supplementary file**

**Performance, computational and degradation mechanistic behavior of novel DAC@PdA@FM MOF nanocomposite for concurrent photocatalytic degradation of cationic and anionic dyes**

**Nora A El-Mahdy^1^, Sayed RH El-Gharkawy^1^ and Magda A Akl*^1^**

**^1^Chemistry Department, Faculty of Science, Mansoura University, Mansoura 35516, Egypt**

**TableS1**: Volumetric titrations of dialdehyde cellulose (DAC) for estimating the average percentage of aldehyde AC%

| **Vcontrol (ml)** | **Vsample (ml)** | **C NaOH (M)** | **m (gm)** | **AC %** | **Average AC%** |
| --- | --- | --- | --- | --- | --- |
| 0.00 | 2.47 | 0.1 | 0.1 | 39.59 | 39.8 |
| 0.00 | 2.50 | 0.1 | 0.1 | 40.07 |  |
| 0.00 | 2.48 | 0.1 | 0.1 | 39.75 |  |

**
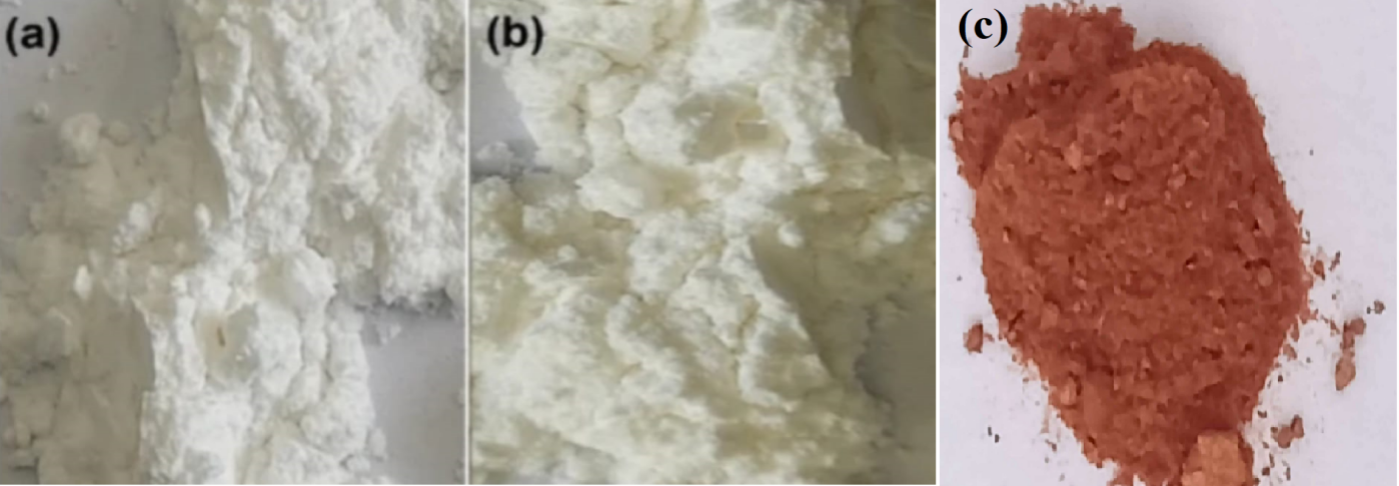
**

**Fig. S1** The digital photographs of (a) native cellulose, (b) DAC, and (c) DAC@PdA.

**Fig.S2.** the biological activity evaluation of DAC@PdA@FM


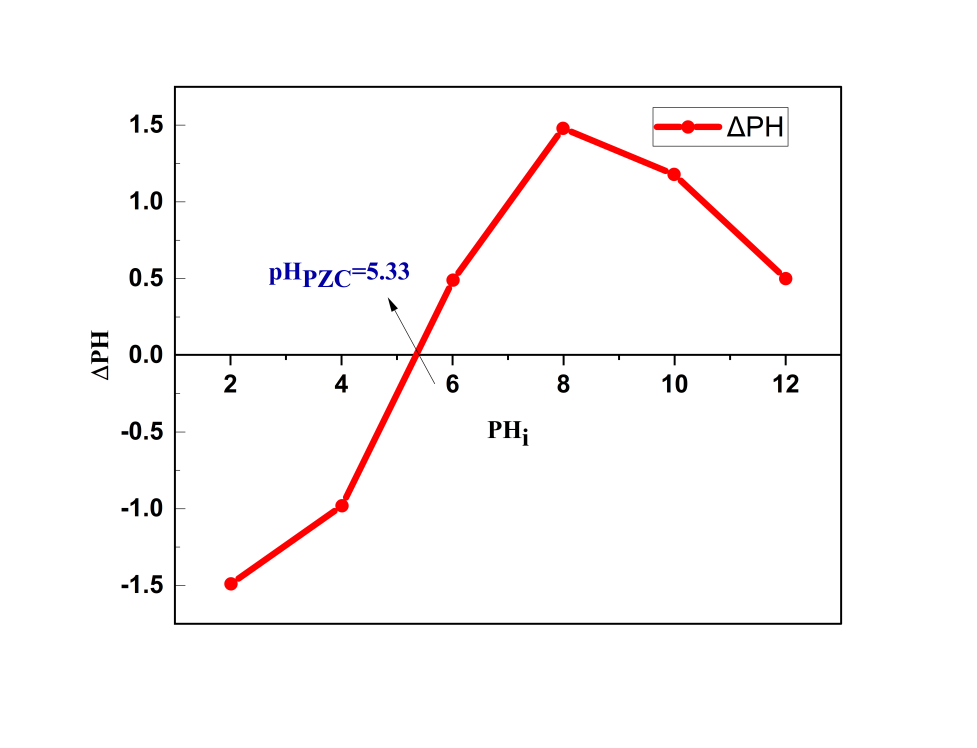


**Fig.S3.** pH_PZC_ of the DAC@PdA@FM.

|  |  |
| --- | --- |
|  | |

**Fig.S4.**Effect of sorbent dose on the degradation efficiency of (TBO, CV and E110) dyes (conditions: 25 ml aqueous solution of 150 mg/L for dyes solution for 30 min at pH 6,8 and 3), respectively**.**

|  |  |
| --- | --- |
|  | |

**Fig.S5**. Effect of dyes initial concentration (conditions: 0.005g of DAC@PdA@FM was taken at optimum pH for each dye for 30 min in range 50 ppm-300 ppm of dyes).

|  |
| --- |

**Fig.S6.** Plot of ln KC vs (1/T) absolute temperature for (TBO, CV and E110) dyes degradation by DAC@PdA@FM.


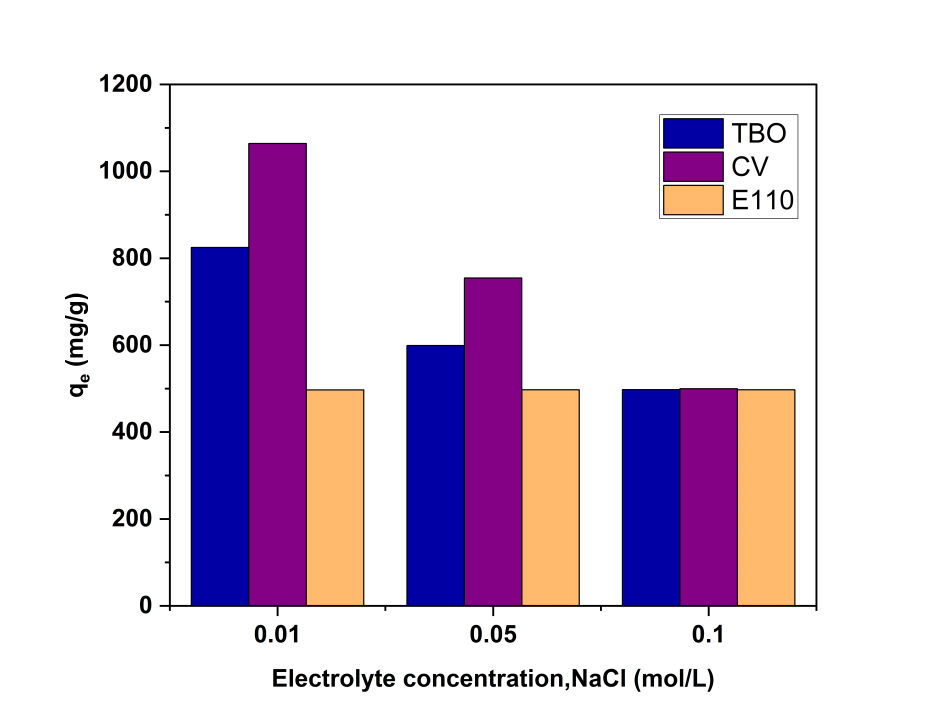


**Fig.S7.** Effect of ionic strength for (TBO, CV and E110) dyes degradation by DAC@PdA@FM (conditions: 0.005g of DAC@PdA@FM was taken at 25 ml 200,250 and 100 ppm of TBO, CV and E110 at optimum pH for 30 min).
